# A Modular Nucleoside Kinase Cascade for the Synthesis of Ribonucleoside Triphosphates

**DOI:** 10.1002/bit.70247

**Published:** 2026-05-24

**Authors:** Oliver T. Damm, Martin Pfeiffer, Johannes Zöhrer, Bernd Nidetzky

**Affiliations:** ^1^ Institute of Biotechnology and Biochemical Engineering Graz University of Technology Graz Austria; ^2^ Austrian Centre of Industrial Biotechnology (acib) Graz Austria

**Keywords:** acetyl phosphate, multistep enzymatic cascade, nucleoside kinase, reaction intensification, ribonucleotide triphosphate synthesis

## Abstract

The increasing demand for affordable nucleoside 5′‐triphosphates (NTPs) necessitates efficient and scalable methods of synthesis. Herein, we report standardized one‐pot enzymatic cascades for the production of all canonical NTPs (ATP, CTP, GTP, UTP). Central to these cascades is the *Saccharomyces cerevisiae* (*Saccharomyces cerevisiae* UMP kinase (*Sc*URA6), which efficiently phosphorylates AMP, CMP, and UMP. ScURA6 is inactive toward GMP, so *Saccharomyces cerevisiae* GMP kinase (*Sc*GMPK) was used to synthesize GTP. NDPs were phosphorylated by *Escherichia coli* acetate kinase (*Ec*AcK), using acetyl phosphate as the phosphate donor in the presence of catalytic amounts of ATP or GTP. Substrate‐specific kinases were used to phosphorylate the nucleoside starting material. The resulting four cascades were optimized for flux through each step to afford the NTP product in the absence of NMP and NDP intermediates. The reactions were intensified for full conversion (≥ 93%) of 50–100 mM substrate within 60–120 min. Alternatively, a modified cascade employing the product NTP as a phosphate shuttle was established, achieving an NTP conversion of ≥ 84%. Preparative‐scale syntheses (10 mL) reached up to 89% isolated yield (≥ 150 mg product), demonstrating a robust and scalable platform for NTP production. The kinases used are active with noncanonical nucleosides and nucleoside analogs, offering flexibility for broad synthetic applicability of the cascade transformations used.

## Graphical abstract

Damm et al. report standardized enzymatic cascades for production of all canonical nucleoside triphosphates from the corresponding nucleosides, using kinase‐catalyzed phosphorylation from acetyl phosphate. Reactions afforded ∼ 100 mM product in near‐quantitative conversion. The kinases used are active with noncanonical nucleosides and nucleoside analogs, offering flexibility for broad synthetic applicability.

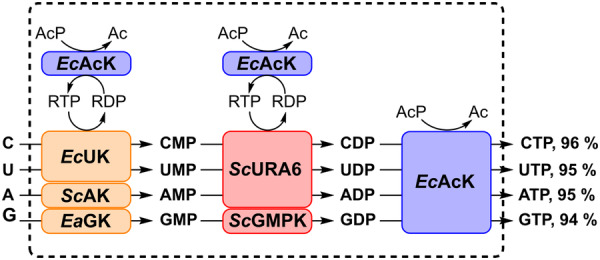

**Abbreviations**
AcK: acetate kinase (EC 2.7.2.1)
AK: adenosine kinase (EC 2.7.1.20)
GK: guanosine kinase (EC 2.7.1.73)
GMPK: guanylate kinase (EC 2.7.4.8)
NDPK: nucleoside 5’‐diphosphate kinase (EC 2.7.4.6)
NK: nucleoside kinase
NMPK: nucleoside 5’‐monophosphate kinase
PPK: polyphosphate kinase (EC 2.7.4.1)
UK: uridine kinase (EC 2.7.1.48)
URA6: uridylate kinase (EC 2.7.4.B1).

## 1 Introduction

Nucleotide 5′‐triphosphates (NTPs) are the building blocks of DNA and RNA and central to signaling and energy transduction (Fontecilla‐Camps 2022; Lane and Fan 2015; Minchin and Lodge 2019). They are indispensable in biotechnology as substrates for nucleic acid synthesis, sequencing, and transcription, as energy carriers in cell‐free biomanufacturing, and as core motifs of therapeutic nucleoside analogs (Hutchison 2007; Li et al. 2024; Meng et al. 2025; Moody et al. 2023; Ricca et al. 2011; Wu et al. 2025; Yadav et al. 2025; Yao et al. 2025). Consequently, efficient, scalable, and standardized methods of NTP synthesis are in high demand.

**Figure 1:**
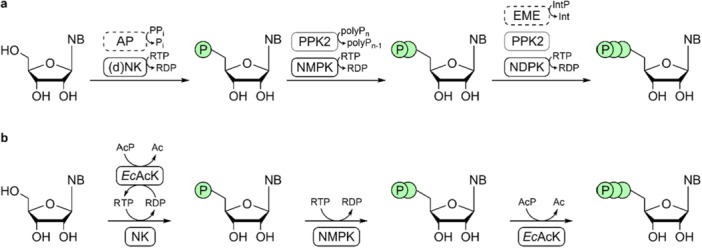
(a) General strategy for (d)NTP synthesis via enzymatic cascade phosphorylation. The scheme lists enzymes capable of catalyzing each of the three reaction steps and their respective phosphate donors (Fehlau et al. 2025). (b) Specific cascade used in this work. The figure indicates the enzyme classes selected for each step and includes the AcK/AcP system for phosphate shuttle regeneration (shown only in the first reaction for clarity). NB, nucleobase; PP_i_, pyrophosphate; P_i_, orthophosphate; RDP/RTP, ribonucleoside di‐/triphosphate; polyP, polyphosphate; IntP/Int, activated phosphate donor (e.g., AcP). AP, acid phosphatase; (d)NK, (2′‐deoxy) nucleoside kinase; EME, energy‐metabolism enzyme (e.g., AcK); NDPK, nucleoside 5′‐diphosphate kinase; NMPK, nucleoside 5′‐monophosphate kinase; (d)NTP, (2’‐deoxy) nucleotide‐5’‐triphosphate; PPK2, polyphosphate kinase 2; P, phosphoryl group.

Established chemical routes exhibit low atom economy and generate substantial amounts of waste (Kaspar et al. 2021; Roy et al. 2016). Biocatalytic cascade phosphorylation can offer a sustainable alternative. In these reactions (Figure 1a), phosphate groups are introduced sequentially on the nucleoside substrate, with each step installing a single phosphoryl unit.

The initial 5′‐phosphorylation can be catalyzed by nucleoside kinases (NKs) or acid phosphatases (APs) (Lane and Fan 2015; Lecoq et al. 2001; Médici et al. 2014; Qian et al. 2014). However, APs require acidic conditions (pH < 5) to suppress phosphate donor hydrolysis, incompatible with downstream kinases optimal at pH 6.0 – 9.5 (Berger et al. 1989; Jong et al. 1993; Lu et al. 2003; Meng et al. 2025; Qian et al. 2014; Usuda et al. 2011; Yoshioka et al. 2014). Integrating all steps in one pot therefore favors an NK‐based design, which is the focus of this study.

The second phosphorylation is typically mediated by nucleoside monophosphate kinases (NMPKs) or polyphosphate kinases (PPK2 II/III) (Berger et al. 1989; Lane and Fan 2015; Matsumoto et al. 2026; Meng et al. 2025; M. Mitton‐Fry et al. 2025; Muüller‐Dieckmann and Schulz 1995; Nocek et al. 2018; Suess and Cornelissen 2026). While many NMPKs are stringent in their substrate use, some eukaryotic homologs show broader tolerance (Muüller‐Dieckmann and Schulz 1995; Xu et al. 2008).

The final phosphorylation to the NTP can follow routes with very different driving forces (Figure 1a): nucleoside diphosphate kinases (NDPKs) are broad in scope but require an NTP donor and operate near equilibrium (*K*_eq_ ≈ 0.6) (Georgescauld et al. 2020; Schaertl et al. 1998). In contrast, energy‐metabolism kinases (e.g., acetate kinase, AcK) use simple donors such as acetyl phosphate (AcP) and strongly favor NTP formation (*K*_eq_ ≈ 10³ − 10⁷) (LoPresti and Cohn 1989; Moffett et al. 2020; Schormann et al. 2019; Spector 1980; Wiseman et al. 2023).

PPK2 enzymes provide an interesting alternative by converting NMPs to NTPs with polyphosphate, either by combining NMP‐ and NDP‐specific PPK2s or using bifunctional PPK2 III (Matsumoto et al. 2026; Motomura et al. 2014). Despite showing broad scope, these systems are only weakly product‐favored (*K*_eq_ ≈ 1.2) and frequently display product dephosphorylation (Frisch et al. 2021; Matsumoto et al. 2026; Meng et al. 2025; Querengässer et al. 2024).

Implementation challenges with polyP include variable solubility of the donor substrate and chain‐length preferences of the PPKs that hinder full conversion and efficient phosphate utilization (Tavanti et al. 2021). In contrast, an NK‐NMPK‐AcK cascade combines a strong driving force (*K*_eq_ ≈ 1500) with a single, readily accessible phosphate donor (AcP) (Spector 1980). Broader application of such a cascade, however, has been limited by the narrow substrate scope of many NMPKs and the observation of reduced reaction efficiency at substrate loadings above 50 mM.

Here, we addressed these gaps by focusing on eukaryotic NMPKs, which are generally more substrate‐flexible than bacterial homologs (Fukui et al. 2025; Segura‐Peña et al. 2004), whereas prior work emphasized the bacterial enzymes (Benčić et al. 2023; Ding et al. 2020; Fehlau et al. 2020). *Saccharomyces cerevisiae* UMP kinase (*Sc*URA6, EC 2.7.4.B1) displays activity toward AMP, CMP, and UMP (Muüller‐Dieckmann and Schulz 1995); *Saccharomyces cerevisiae* GMP kinase (*Sc*GMPK, EC 2.7.4.8) was selected for GMP (Berger et al. 1989). *Escherichia coli* AcK (EC 2.7.2.1) served to establish the terminal phosphorylation module due to its demonstrated ATP/GTP regeneration from AcP (Figure 1b) (Pfeiffer et al. 2025), with acetate as the sole by‐product (Ruccolo et al. 2022; Siedentop et al. 2023).

The multienzyme cascades converge at the NMP and NDP phosphorylation steps (Figure 1b). Substrate‐specific NKs were used for the initial phosphorylation, as no reasonably universal NK has been reported in literature so far: *E. coli* uridine kinase (*Ec*UK, EC 2.7.1.48) for uridine (U) and cytidine (C); *Saccharomyces cerevisiae* adenosine kinase (*Sc*AK, EC 2.7.1.20) for adenosine (A); and *Exiguobacterium acetylicum* guanosine–inosine kinase (*Ea*GK, EC 2.7.1.73) for guanosine (G). NKs were chosen based on reported overexpression in *E. coli*, high activity and suitable operational stability (Benčić et al. 2023; Kawasaki et al. 2000; Pfeiffer et al. 2025; Zhao et al. 2024).

Using this basic NK‐NMPK‐AcK architecture (Figure 1b), we developed standardized cascades based on *Sc*URA6 (or *Sc*GMPK) and *Ec*AcK. Comprehensive characterization enabled unified conditions that suppressed intermediate buildup and supported 50 – 100 mM NTP formation. Scale‐up to 10 mL delivered ≥ 150 mg of NTP at > 93% conversion and up to 76% isolated yield. These results demonstrate that modular kinase cascades enable rapid, efficient, and scalable NTP production with high space‐time yields.

## 2 Materials and Methods

### 2.1 Materials

A and U were from Carl Roth GmbH (Karlsruhe, Germany). G was from Sigma Aldrich (St. Louis, MO, USA). **C**, all NMPs, NDPs and NTPs were from Biosynth (Staad, Switzerland). The enzymes used were *Ec*UK (Uni‐Prot accession number: P0A8F4), *Ec*AcK (Uni‐Prot: P0A644), *Sc*AK (UniProt: P47143), *Sc*GMPK (EC 2.7.4.8, UniProt: P15454), *Sc*URA6 (UniProt: P15700) and *Ea*GK (Uni‐Prot: O24767). Gene fragments encoding *Ea*GK, *Sc*AK and *Sc*GMPK were ordered from GenScript (Rijswijk, Netherlands). *E. coli* NEB 10‐beta and NiCo21 (DE3) strains were from New England Biolabs GmbH (Ipswich, MA, USA) and the pET‐28a(+) vector was from E. Merck KG (Darmstadt, Germany). AcP was synthesized according to Tasnádi et al. (2018).

### 2.2 Cloning, Expression and Purification

Expression strains for *Ec*UK, *Sc*URA6 and *Ec*AcK (*E. coli* BL21(DE3) pET‐15b(+)) were available from Pfeiffer et al. (2025). Synthetic genes encoding *Ea*GK, *Sc*AK and *Sc*GMPK were individually cloned into pET‐28a(+) (Supporting Information). Plasmids were amplified in *E. coli* NEB 10‐beta; expression was done in *E. coli* NiCo21 (DE3). Proteins were purified by Ni‐NTA affinity chromatography using standard protocols (Supporting Information). Purity was assessed by SDS‐PAGE (Supporting Information Figure S1).

### 2.3 Specific Activity Determination

Unless stated otherwise, specific activities (*A*_s_) were measured at 30°C in 50 mM TAPS (pH 8.0, 1.0 mL volume, 1.5 mL reaction tube) with agitation at 300 rpm (ThermoMixer C, Eppendorf, Hamburg, Germany). The pH was measured at each sampling point and at regular intervals between samples (Metrohm 691 pH meter, Metrohm, Herisau, Switzerland). It was maintained between 7.8 and 8.2 by addition of 0.5 µL of 5 M NaOH. Reactions were initiated by adding a premixed enzyme solution (1%–10% vol/vol) to temperature‐equilibrated, pH‐adjusted substrate solutions. Each enzyme was assayed within the full NK‐NMPK‐AcK cascade by supplying the enzyme under study in limiting amounts and downstream enzymes in excess (0.1 mg mL⁻¹). Standard mixtures contained 10 mM substrate, 0.5 mM ATP (for G and U) or GTP (for A and C), 40 mM AcP, and 10 mM MgCl₂. Samples (60 µL) were withdrawn over 60 min, quenched 1:2 with methanol, centrifuged (30 min, 21300*g*) to remove any particles, and analyzed by RP‐HPLC.

*Ec*UK and *Sc*AK were characterized using *Sc*URA6 as the NMPK. *Ec*UK was used at 0.00025 mg mL^−1^ and *Sc*AK at 0.005 mg mL^−1^. *Ea*GK, paired with *Sc*GMPK, was analyzed at 0.07 mg mL^−1^. *Sc*URA6 was evaluated at 0.0005 mg mL^−1^ (AMP) and 0.001 mg mL^−1^ (CMP, UMP). *Sc*GMPK was tested at 0.0005 mg mL^−1^. *Ec*AcK was assayed at 0.0001 mg mL^−1^ (GDP, UDP), at 0.0002 mg mL^−1^ (ADP), and at 0.005 mg mL^−1^ (CDP) in the absence of NK or NMPK. The corresponding *A*_s_ values were calculated from the linear phase of product formation with time. One unit (U) of activity is defined as the amount of enzyme required to convert 1 µmol of substrate min^−1^ under the specified reaction conditions described above.

### 2.4 Enzymatic Cascade Phosphorylation

Unless specified, cascade reactions were performed at 30°C in 50 mM TAPS (pH 8.0, 1.0 mL scale in a 1.5 mL reaction tube) with pH control as established in Section 2.3 (7.8–8.2) and 300 rpm agitation. Substrate loadings were 10, 25, 50, and 100 mM. ATP (for G and U) or GTP (for A and C) was added at 5 mol%; AcP was used in fourfold molar excess over the substrate; MgCl₂ was supplied equimolar to substrate up to 100 mM. Sampling followed the procedure in Section 2.3. For the A and G cascades, the dissolved nucleoside/nucleotide concentrations were also measured spectrophotometrically; samples were centrifuged (5 min, 21300*g*) and supernatants diluted to 0.5 mM nucleoside/nucleotide.

### 2.5 Preparative Synthesis of NTPs

Reactions (10 mL; 25 mL glass beaker, 2.5 cm diameter) were run at 30°C in 50 mM TAPS (pH 8.0) with 300 rpm magnetic stirring (10 mm × 3 mm stir bar). The temperature was set by placing the beaker in a temperature‐controlled water bath. Temperature and stirring rate were controlled with a Magnetic stirrer MR 3001 K unit (Heidolph Instruments GmbH & Co. KG, Schwabach, Germany). The pH was monitored continuously with a pH electrode placed into the reaction mixture. It was controlled at a value of 8.0 using 5 M NaOH that was added in portions of 5 µL.

Nucleosides were 50 mM with 5 mol% ATP (G and U) or GTP (A and C), 200 mM AcP, and 50 mM MgCl₂. *Ec*UK, *Sc*AK, and *Ea*GK were 2, 1.7, and 5 U mL⁻¹, respectively; NMPK and *Ec*AcK were 17 and 100 U mL⁻¹. Progress was monitored by RP‐HPLC and UV spectroscopy; NaOH addition was recorded.

Enzymes were removed by ultrafiltration (Vivaspin Turbo 15 RC ultrafiltration concentrators 10 kDa cutoff; Sartorius, Göttingen, Germany, 3095*g*, 4°C). Products were purified by AEX on an ÄKTA Go (Cytiva, Marlborough, MA, USA) with a XK 26/400 column packed with DEAE Sepharose Fast Flow (125 mL, Cytiva). Diluted crude product (50 mL, 10 mM nucleoside/nucleotide) was loaded, unbound material washed with water, and product eluted with 300 mM NH₄HCO₃ at 40% (120 mM) followed by step increases to 100%. Product fractions were concentrated (30°C, 30 mbar, 150 rpm, Laborota 4000 efficient rotary evaporator, Heidolph Instruments GmbH & Co. KG, Schwabach, Germany), lyophilized (Christ Alpha 1‐4 LSCplus freeze dryer, Martin Christ Gefriertrocknungsanlagen GmbH, Osterode am Harz, Germany), and characterized by RP‐HPLC and ¹H, ¹³C, and ³¹P NMR. Purity was determined by RP‐HPLC.

### 2.6 Auto‐Regeneration Cascades

Auto‐regeneration cascades were defined as reactions in which the target NTP was added in catalytic amounts and continuously regenerated by *Ec*AcK. Reactions (1.0 mL) contained 50 mM nucleoside, 200 mM AcP, 50 mM MgCl₂, and 2.5 mM target NTP in 50 mM TAPS (pH 8.0) at 30°C, with ThermoMixer agitation at 300 rpm (C, U) or 1000 rpm (A, G). Enzyme loadings matched the standard 1:10:60 activity ratio for A and U; for C, enzyme concentrations were doubled; for G, tripled. Conditions, sampling, and analytics followed Sections 2.3–2.4.

### 2.7 Synthesis of Pseudouridine (Ψ)−5'‐triphosphate and Analogs Thereof

Pseudouridine‐5’‐monophosphate (ΨMP) and the analogs 2‐thio‐ΨMP (2t‐ΨMP), 6‐amino‐ΨMP, 5‐β‐d‐arabinofuranosyl‐ΨMP (araΨMP), and 5‐β‐d‐xylofuranosyl‐ΨMP (xylΨMP) were prepared as described by Ribar et al. (2024). The corresponding 5′‐triphosphates were then generated by phosphorylation with *Sc*URA6 and *Ec*AcK at the same enzyme concentrations, using 5 mol% ATP as the phosphate shuttle and 3 equivalents of AcP (Supporting Information Figure S15). Reactions were conducted with 10–50 mM substrate in 50 mM TAPS (pH 8.5) supplemented with 10 mM MgCl_2_ at 30°C and 650 rpm.

### 2.8 Reversed‐Phase (RP)‐HPLC

Samples (10 µL) were analyzed on a Kinetex 5 µm C18 (100 Å) column (Phenomenex Ltd., Aschaffenburg, Germany) at 40°C on a Shimadzu LC‐20 with UV detection at 262 nm (Shimadzu Europe GmbH, Duisburg, Germany). Separation was performed in isocratic flow using 84% potassium phosphate buffer (20 mM; pH 5.9) with tetrabutylammonium bromide (40 mM) and 16% acetonitrile. Data were processed using LabSolutions CS software (version 5.110). Typical retention times are listed in Supporting Information Table S1.

### 2.9 Concentration Determination

Protein concentrations were determined by absorbance at 280 nm using molar extinction coefficients calculated with ExPASy ProtParam: *Ea*GK (10095 M⁻¹·cm⁻¹; 32.5 kDa), *Ec*UK (17085 M⁻¹·cm⁻¹; 29.3 kDa), *Sc*AK (26025 M⁻¹·cm⁻¹; 26.0 kDa), *Sc*GMPK (14440 M⁻¹·cm⁻¹; 20.8 kDa), *Sc*URA6 (6210 M⁻¹·cm⁻¹; 22.9 kDa), and *Ec*AcK (25245 M⁻¹·cm⁻¹; 43.3 kDa) (Walker 2005).

Dissolved concentrations of A, G, and their nucleotides were determined by absorbance at 260 nm using experimental extinction coefficients for ATP (14.8 mM⁻¹·cm⁻¹) and GTP (11.5 mM⁻¹·cm⁻¹). C and U concentrations in pure preparations were obtained using literature values of 9 mM^−1^ cm^−1^ and 9.2 mM^−1^ cm^−1^ (Pitha et al. 1968; Plum 2000).

## 3 Results and Discussion

### 3.1 Enzyme Production and Characterization

All six enzymes were produced at high purity (Supporting Information Figure S1) with expression levels ≥ 60 mg purified protein L^−1^ of *E. coli* culture. Their *A*_s_ values were determined within the NK‐NMPK‐AcK cascade under standard conditions (30°C, pH 8.0), informing activity‐balanced cascade design (Table 1). This design allowed us to tune enzyme ratios based on pathway context and to identify potential flux bottlenecks caused by stepwise imbalances.

**Table 1:** Key parameters of the biocatalysts used.

Parameters	*Ea*GK	*Ec*UK	*Sc*AK	*Sc*GMPK	*Sc*URA6	*Ec*AcK
*A*_s_ [U/mg]^a^						
A	n.d.^d^	n.d.	15 ( ± 1.2)	n.d.	8.2 ( ± 0.9) × 10^2^	3.8 ( ± 0.6) × 10^3^
C	n.d.	1.1 ( ± 0.2) × 10^2^	n.d.	n.d.	3.3 ( ± 0.6) × 10^2^	4.1 ( ± 0.5) × 10^2^
G	12 ( ± 0.4)^e^	n.d.	n.d.	3.2 ( ± 0.8) × 10^2^	n.d.	7.0 ( ± 0.2) × 10^3^
U	n.d.	5.6 ( ± 0.4) × 10^2^	n.d.	n.d.	3.2 ( ± 0.2) × 10^2^	4.5 ( ± 0.5) × 10^3^
Yield [mg/L]^b^	~70	83 ± 12	∼60	∼70	133 ± 40	~100
Temp. [°C]^c^	26–39 (Mori et al. 1997)	37 (Qian et al. 2014)	37 (Lu et al. 2003)	30 (Nomura et al. 2014)	37 (Xu et al. 2008)	30 (Yoshioka et al. 2014)
pH^c^	7.5 (Usuda et al. 2011)	7–8 (Qian et al. 2014)	8 (Lu et al. 2003)	7.7 (Berger et al. 1989)	6–9.5 (Jong et al. 1993)	6.5–7.5 (Yoshioka et al. 2014)

^a^ Enzymes were used as purified protein preparations.

^b^ Protein yield per volume of *E. coli* production culture after purification. Standard deviation (S.D.) is estimated for *N* = 2.

^c^ Optimum temperature and pH from literature.

^d^ n.d., not detectable (detection limit: < 5 × 10^‐2^ U mg^‐1^).

^e^ SD were determined from the residual deviations of data points within the linear phase (N ≥ 4) relative to the corresponding linear regression fit.

*Ea*GK and *Sc*AK exhibited *A*_s_ values ~ 7.5‐ and ~37‐fold lower, respectively, than *Ec*UK toward **C** and **U**. *Ec*AcK was the most active enzyme in the set, exceeding 10^3^ U mg^−1^ with ADP, GDP and UDP. *Sc*URA6 showed comparatively high *A*_s_ toward AMP, CMP, and UMP but was inactive toward GMP under assay conditions, consistent with prior structural and biochemical evidence (Muüller‐Dieckmann and Schulz 1995). Substitution with *Sc*GMPK restored efficient phosphorylation of GMP to GDP, with an *A*_*s*_ value comparable to those of *Sc*URA6 for CMP/UMP, thereby maintaining a harmonized flux profile for the NMP → NDP step across substrates.

Using *Sc*URA6 and *Sc*GMPK, all canonical NTPs were accessible. Representative assays (Supporting Information Figures S2–S4) reached ≥ 2 mM NTP depending on the limiting enzyme. Although optimized for activity measurement rather than production, these results underscore the platform's broad scope. The flexible substrate specificity of *Sc*URA6, relative to many established NMPKs, makes it a particularly versatile and valuable biocatalyst for NTP synthesis (Fehlau et al. 2025; Muüller‐Dieckmann and Schulz 1995; Pfeiffer et al. 2025).

The crystal structure of *Sc*URA6 (PDB ID: 1UKY; Muüller‐Dieckmann and Schulz 1995) indicates a flexible nucleobase‐binding pocket. Based on binding‐pocket analysis (Supporting Information Figure S5a−c), we hypothesize that the enzyme can accommodate adenine and the smaller uracil and cytosine as substrates. GDP binding in *Sc*URA6 (Supporting Information Figure S5d) would however be disfavored by steric hindrance associated with the comparably large guanine group. In contrast, GMP fits well within the active site of *Sc*GMPK (Supporting Information Figure S6), which is consistent with the observed substrate specificity.

### 3.2 Cascade Phosphorylation for NTP Synthesis

#### 3.2.1 Pyrimidine NTP Cascades

##### 3.2.1.1 General Considerations

We aimed to establish broad‐scope cascades delivering high conversions at elevated substrate loadings (50–100 mM), at least an order of magnitude above most prior reports (Benčić et al. 2023; Fehlau et al. 2020; Meng et al. 2025). At these concentrations, continuous pH control is essential, enzyme activities must be balanced to prevent intermediate accumulation, and Mg²⁺ supply can easily become limiting because it is required in near stoichiometric amounts.

##### 3.2.1.2 Enzyme Loading and Activity Ratio

**Figure 2:**
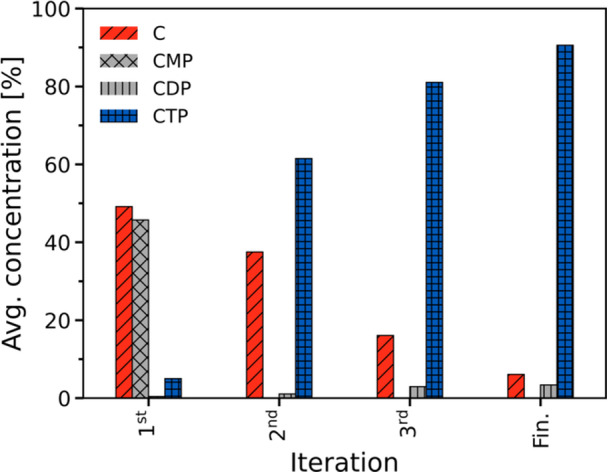
Iterative process of cascade reaction optimization for CTP synthesis. For each iteration, the endpoint composition (percent of each nucleoside/nucleotide) was quantified by RP‐HPLC and averaged across all reactions in that iteration (1st and 2nd iteration: N = 2; 3rd and final iteration: *N* = 4). Reaction conditions: 1 mL, 50 mM TAPS (pH 8.0), 30°C, 300 rpm. Iteration‐specific adaptations are detailed in Section 3.2‐1; enzyme loadings are provided in the Supporting Information (Supporting Information Figures S7–S9).

Initial experiments at 10 and 25 mM C (1st iteration; see Figure 2) employed equal volumetric activities of *Ec*UK and *Sc*URA6, with *Ec*AcK at four‐fold excess. The limiting enzyme activity was scaled with substrate (10 mM: 0.17 U mL⁻¹; 25 mM: 0.40 U mL⁻¹), such that, based on calculated net rates, complete conversion to CTP would be expected within ~120 min.

Contrary to expectation, only ~5% CTP formation was observed after 180 min, with substantial CMP accumulation (~50%, Supporting Information Figure S7a–b). These results clearly indicate a kinetic bottleneck at the CMP phosphorylation step. Increasing *Sc*URA6 and *Ec*AcK activity ten‐fold while maintaining the original *Ec*UK loading (2nd iteration; Figure 2) relieved this limitation and significantly improved CTP formation, yielding 51% (10 mM) and 71% (25 mM) conversion after 180 min (Supporting Information Figure S7c,d). Nevertheless, complete conversion, which was anticipated given the strong thermodynamic drive from AcP consumption, remained unattained. We hypothesized that an unfavorable enzyme‐to‐substrate mass ratio (0.013 mg enzyme to 2.4 mg substrate) limited effective turnover. We therefore further increased total enzyme loading ∼2.5‐fold (activity ratio *Ec*UK:*Sc*URA6:*Ec*AcK = 1:10:40‐60, 3rd iteration; Figure 2).

##### 3.2.1.3 Mg^2+^ Requirement

Nucleoside/nucleotide kinases require Mg^2+^ for enzyme activity. Mg^2+^ coordination to the NTP phosphate groups is critical for substrate binding recognition and plays a key role in positioning for catalysis. While Mg^2+^ is an enzyme cofactor that is not consumed in the reaction, its complexation in solution by the NTP product released must be considered. Mg^2+^ solubility shows a complex dependence on concentration in the presence of phosphate and phosphate group‐containing compounds. The initial concentration of Mg^2+^ used therefore becomes an important process parameter to be targeted for optimization. MgCl_2_ was provided at stoichiometric amounts relative to the nucleoside substrate concentration used, in accordance with literature (Lassila et al. 2011).

At substrate concentrations of 10, 25, and 50 mM, conversions up to 95% were obtained within 240 min. However, increasing the C concentration to 100 mM reduced the conversion to ~54% over the same period (Supporting Information Figure S7), and formation of a white solid (most likely Mg(OH)_2_) was observed upon NaOH addition for pH control. Neither extended reaction times nor increased enzyme loading restored activity.

We concluded tentatively that under the conditions used, Mg^2+^ could become limiting due to its precipitation during the reaction. Therefore, the 100 mM C reaction was repeated with a lowered Mg^2+^ concentration of 50 mM. Precipitation was suppressed and high conversion was restored (> 90% after 240 min; Supporting Information Figure S8). Notably, initial NTP formation rates were comparable at 50 and 100 mM Mg²⁺. The Mg^2+^ concentration was therefore limited to 50 mM in all subsequent reactions (final iteration; Figure 2).

**Figure 3:**
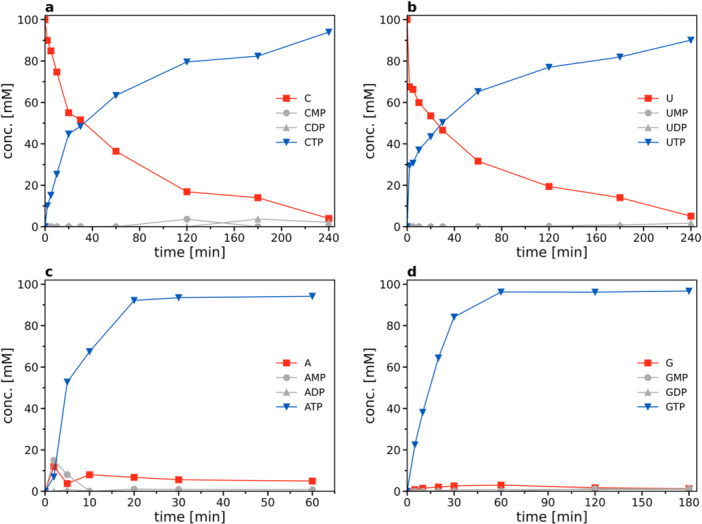
Cascade reactions for flexible NTP synthesis from 100 mM nucleoside substrate. General conditions: 1.0 mL total volume, 50 mM TAPS (pH 8.0), 30°C, 50 mM MgCl₂, 5 mol% ATP (for G, U) or GTP (for A, C), and 200 mM AcP. Agitation at 300 rpm (C, U) or 1000 rpm (A, G) in an Eppendorf TherMomixer C. Panel‐specific enzyme loadings and cumulative NaOH addition: a 10 U mL^−1^ *Ec*UK, 33 U mL^−1^ *Sc*URA6, 200 U mL^−1^ *Ec*AcK, 28.5 µL 5 M NaOH. b 3 U mL^−1^ *Ec*UK, 33 U mL^−1^ *Sc*URA6, 200 U mL^−1^ *Ec*AcK, 31.5 µL 5 M NaOH. c 3.3 U mL^−1^ *Sc*AK, 33 U mL^−1^ *Sc*URA6, 200 U mL^−1^ *Ec*AcK, 23.5 µL 5 M NaOH. d 10 U mL^−1^ *Ea*GK, 33 U mL^−1^ *Sc*GMPK, 200 U mL^−1^ *Ec*AcK. 28.5 µL 5 M NaOH. Values are dilution‐corrected to account for NaOH addition.

High CTP conversions were achieved across all substrate concentrations (Supporting Information Figure S9). At 100 mM (discussed as a representative example, Figure 3a), near quantitative conversion (~94%) was achieved within 240 min, with CMP and CDP at ~6% in the final product mixture. The reaction generated approximately ∼91 mM CTP after accounting for dilution during pH control.

This clean substrate‐to‐product conversion of C into CTP demonstrates effective flux regulation: the first phosphorylation step is rate‐limiting, while downstream steps proceed faster, suppressing intermediate accumulation. A graph summarizing the results of the different iterations of the cytidine cascade can be seen in Figure 2; the underlying data are provided in Supporting Information Table S3.

##### 3.2.1.4 Transfer to UTP Synthesis

Because *Ec*UK exhibits a five‐fold higher *A*_s_ toward U than C (Table 1), we anticipated direct transfer of the optimized scheme to UTP synthesis with the *Ec*UK–*Sc*URA6–*Ec*AcK cascade. Applying the same conditions used for the C cascade afforded ~93% conversion of 100 mM U to ∼90 mM UTP (dilution corrected) within 240 min (Figure 3b) with consistent behavior at 50 mM U (Supporting Information Figure S10). These results demonstrate the seamless transferability of the *Ec*UK–*Sc*URA6–*Ec*AcK cascade for synthetic application to pyrimidine NTPs.

#### 3.2.2 Purine NTP Cascades

##### 3.2.2.1 Integration of Cascade Design With Reaction Engineering

Unlike pyrimidine nucleosides, A and G exhibit low aqueous solubility (A: < 25 mM; G: < 1 mM; 25°C), further diminished under high‐solute conditions (Human Metabolome Project 2021a, 2021b). Consequently, switching from well‐soluble substrates (C and U) required a strategy to overcome the substrate solubility limitations. To achieve 100 mM NTP, we addressed solubility without introducing cosolvents (e.g., DMSO), which can compromise multienzyme stability and complicate optimization. Instead, A and G were used as suspended solids, and the agitation rate increased to 1000 rpm (vs 300 rpm standard; ThermoMixer) to enhance solid‐liquid mass transfer (Tokura et al. 2019).

However, the analytical protocols were adapted accordingly to account for the presence of solid substrate: total dissolved A‐ or G‐derived species were quantified spectrophotometrically and combined with RP‐HPLC to obtain accurate conversions.

*Sc*AK and *Ea*GK are specific kinases for A and G, respectively, and have lower *A*_s_ values than *Ec*UK with C or U (Table 1). Nonetheless, with adjusted enzyme loadings, and substituting *Sc*URA6 with *Sc*GMPK for G, we established functional ATP and GTP cascades. Indeed, production of ATP from 100 mM A reached ~94% conversion, corresponding to ~92 mM ATP in the crude product solution, within 60 min, with negligible AMP/ADP accumulation (Figure 3c). The soluble concentration of A initially exceeded 10 mM, indicating that A consumption was slower than its solubilization under these conditions. Thus, increased agitation rate was the only modification required to adapt the design.

##### 3.2.2.2 Adaptation of the Cascade for GTP Synthesis

For GTP synthesis, *Sc*URA6 was replaced by *Sc*GMPK to enable GMP phosphorylation. In this system, enhanced agitation proved particularly critical, as RP‐HPLC analysis indicated that the overall conversion was governed by G dissolution kinetics.

With a detection limit below ∼1.2 × 10^−3 ^mM for each species under the analytical conditions used, GTP was the only detectable compound during most of the reaction (Supporting Information Figure S11). G was undetectable initially and remained very low later (< 1 mM; Figure 2d). From the initial GTP formation rate, the G solubilization rate was estimated as ∼2 mM min⁻¹. GTP production was also shown at lower G loadings (Supporting Information Figure S12), with the time to reach the targeted conversion (≥ 90%) increasing and the GTP formation rate decreasing with lower G concentrations due to limited soluble G available for *Sc*GMPK. Despite this perceived limitation, the reaction reached ~97% conversion of 100 mM G to 94 mM GTP (dilution corrected) within 180 min.

### 3.3 Preparative Synthesis of NTPs

#### 3.3.1 Scale‐Up of Cascade Reactions

Having established robust conditions for all NTP cascades, their scalability was evaluated by increasing the reaction volume from 1.0 to 10 mL. Agitation was switched from a Thermomixer to magnetic stirrer at a nominal 300 rpm, which was shown in preliminary experiments to promote efficient solubilization of A and G. Nucleoside concentrations were fixed at 50 mM, with all other parameters identical to those used at small scale.

**Figure 4:**
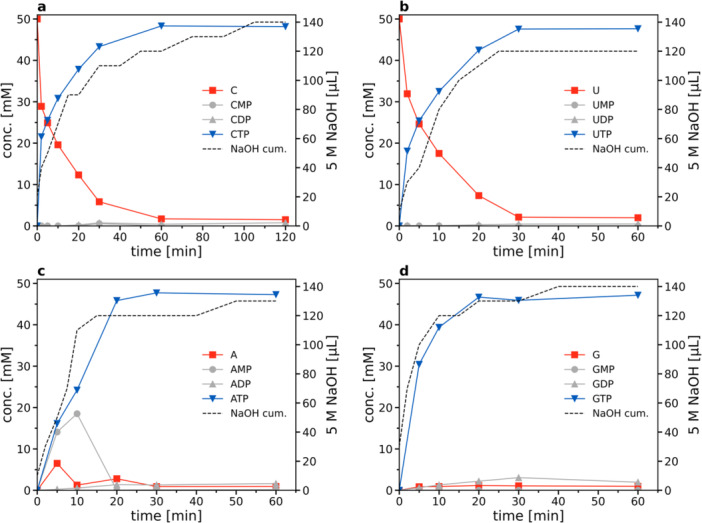
Preparative NTP synthesis in 10 mL reactions. General conditions: 50 mM TAPS (pH 8.0), 30°C, 300 rpm (magnetic stirring), 50 mM MgCl₂, 5 mol% ATP (for G, U) or GTP (for A, C), and 200 mM AcP. Panel‐specific enzyme loadings and cumulative NaOH addition: a 2.5 U mL^−1^ *Ec*UK, 17 U mL^−1^ *Sc*URA6, 100 U mL^−1^ *Ec*AcK, 140 µL 5 M NaOH. b 2.5 U mL^−1^ *Ec*UK, 17 U mL^−1^ *Sc*URA6, 100 U mL^−1^ *Ec*AcK, 120 µL 5 M NaOH. c 1.7 U mL^−1^ *Sc*AK, 17 U mL^−1^ *Sc*URA6, 100 U mL^−1^ *Ec*AcK, 130 µL 5 M NaOH. d 5 U mL^−1^ *Ea*GK, 17 U mL^−1^ *Sc*GMPK, 100 U mL^−1^ *Ec*AcK, 140 µL 5 M NaOH. Values are dilution‐corrected to account for NaOH addition.

Both C and U were fully converted (≥ 95%) to their corresponding NTPs within 60 min and 30 min, respectively (Figure 4a–b). The purine cascades also scaled effectively, reaching ≥ 94% within 20–30 min (Figure 4c–d). All reactions exhibited comparable kinetic profiles and, notably, no accumulation of cascade intermediates.

Incomplete substrate utilization is a common challenge in enzyme cascades, often yielding mixtures of NMP, NDP, and NTP which substantially reduces overall conversion (Matsumoto et al. 2026; Meng et al. 2025; Suess and Cornelissen 2026). This is often observed when using PPKs (Benčić et al. 2023; Matsumoto et al. 2026; Meng et al. 2025). In these cases, adding AcK can shift equilibria toward NTP formation (Meng et al. 2025). Accumulation of intermediates may also result from an insufficient supply of co‐substrates; maintaining a constant co‐substrate level through increased loading or in situ recycling has been shown to reduce such buildup (Fehlau et al. 2020).

During scale‐up, NaOH demand increased and was monitored in real time as process metric. Because nucleoside and AcP conversion follows a strict 1:3 stoichiometry, both the total NaOH volumes added (120 µL – 140 µL of 5 M NaOH) and addition profiles were highly consistent across all reactions (Figure 4). Rapid conversion (≥ 94% within 60 min or less) minimizes concerns about AcP hydrolysis. Siedentop et al. (2023) reported ~30% AcP hydrolysis after 5 h at 20°C with 20 mM MgCl₂ and ~100% after 5 h at 50°C. However, given the rapid turnover here, AcP degradation was negligible, and only a slight excess of the phosphate donor (1 equiv.) was required.

Earlier work by Pfeiffer et al. (2025) has investigated the operational stability of *Sc*Ura6 and *Ec*AcK under conditions (AcP 1.15 M, MgCl_2_ 30 mM; pH control) largely comparable to the ones used here. A total turnover number (TTN) of 2.2 × 10^7^ (*Sc*Ura6) and 3.6 × 10^6^ was determined for *Sc*Ura6 and *Ec*AcK, respectively. For both enzymes, the turnover number (TON) in the synthesis reactions conducted here was lower by least one order of magnitude than the literature‐reported TTN. The TON calculated as the average over all four cascade reactions was 2.8 × 10^4^ for *Sc*Ura6 and 5.0 × 10^4^ for *Ec*AcK. We concluded therefore that *Sc*Ura6 and *Ec*AcK stability was probably not a factor limiting the substrate conversion under the conditions used here. However, although not examined in detail here, enzyme operational stability is recognized as an important topic for development and optimization.

The conditions established here provide a robust starting point for processes employing the same or closely homologous enzymes and are expected to be broadly applicable. Reaction parameters (temperature and pH) should be adjusted to the enzyme‐specific optima. Mg²⁺ supplied at an equimolar concentration relative to the substrate (up to 50 mM) constitutes a practical initial condition. Under these settings, enzyme loading remains the primary parameter requiring systematic optimization.

#### 3.3.2 Integration With Product Isolation

Post reaction, the crude reaction mixture contained ~36% NTP relative to total dissolved species in 10 mL. NTPs were purified from residual substrates (mainly AcP) and byproducts via AEX chromatography using volatile NH_4_HCO_3_, which was removed by evaporation.

**Figure 5:**
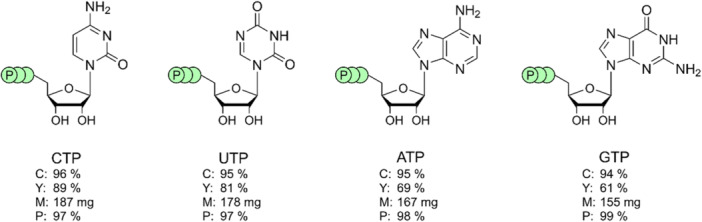
Summary of NTP production. C, conversion; Y, isolated yield, M, mass isolated; P, purity. *N* = 1.

High isolated yields (calculated as the mass of isolated product divided by the theoretical maximum mass) and high purities were achieved, as confirmed by RP‐HPLC: CTP, 187 mg (89%, 97% purity); UTP, 178 mg (81%, 97% purity); ATP, 167 mg (69%, 98% purity), and GTP, 155 mg (61%, 99% purity). An overview is provided in Figure 5; corresponding ^1^H, ^13^C and ^31^P NMR spectra are shown in Figures N1–N8.

We note that the purification method used yielded products of high purity, at the expense of isolated yield. Yield losses primarily arose from limited chromatographic resolution between NTP and NDP peaks (Supporting Information Figure S13). To maintain high purity, early fractions containing trace NDP were discarded. ATP and GTP posed additional challenges due to later elution and tailing, necessitating removal of late fractions to avoid cross contamination, particularly given their similar retention times.

#### 3.3.3 A Generalizable Framework for Auto‐Regenerative Nucleotide Synthesis

**Figure 6:**
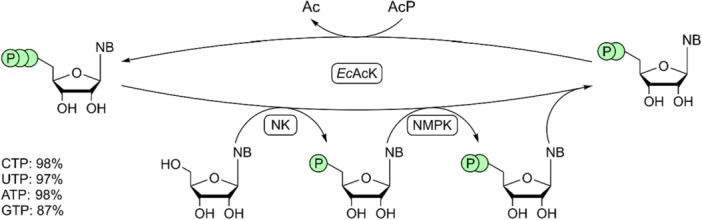
NTP synthesis using auto‐regeneration cascades. The graph shows the final conversion reached for each NTP. Detailed results are shown in Supporting Information Figure S14.

To streamline isolation, we implemented auto‐regenerative cascades in which the target NTP is seeded in catalytic amounts and continuously recycled in situ by AcK, eliminating auxiliary NTPs and preventing their co‐elution with product (Figure 6).

At 1 mL scale and 50 mM substrate, the A, C, and U cascades reached ≥ 96% conversion within 90–180 min without detectable intermediate buildup. For C, doubling the enzyme loading sustained high flux. For G, a threefold increase in enzyme loading delivered 87% conversion at 180 min with only 12% GDP accumulation (Supporting Information Figure S14).

Collectively, these results establish auto‐regeneration as a robust, modular platform for RNA nucleotide synthesis. By using the product NTP itself as the phosphate shuttle, the cascades remove extraneous nucleotide species and therefore streamline purification. The cascade transformations are readily extendable to 2’‐deoxy‐nucleoside triphosphates (dNTPs) and other valuable nucleoside analogs, broadening their synthetic scope.

#### 3.3.4 Advance in the Biocatalytic Synthesis of NTPs

Fully flexible phosphorylation cascades remain uncommon despite numerous target‐specific reports (Benčić et al. 2023; Da Costa et al. 2007; Eltoukhy and Loderer 2022; Fehlau et al. 2020; Frisch et al. 2021; Hennig et al. 2007; Matsumoto et al. 2026; Meng et al. 2025; Scott et al. 2004). To contextualize our platform, we compared it with four representative multi‐substrate systems: an NK‐NMPK‐NDPK cascade with pyruvate kinase/phosphoenolpyruvate recycling that converts A, C, and several analogs thereof (Fehlau et al. 2020); a polyphosphate‐driven NK‐PPK2 II‐PPK2 I route for all four ribonucleotides (Benčić et al. 2023); an AP‐PPK2 III‐AcK design for 2′‐methoxyethyl‐ and 2′‐fluoro‐ATP (Meng et al. 2025); and a bifunctional polyphosphate kinase platform for the synthesis of NTPs during in vitro transcription (Matsumoto et al. 2026).

While prior platforms achieved up to 98% conversion, our NK‐NMPK‐AcK cascades operated ~10–20‐fold faster (≤ 2 h vs 19–44 h), used ~ 2–7‐fold less co‐substrate and ~17–37‐fold less total enzyme, and delivered ~300–800‐fold higher space‐time yield (24 vs 0.03–0.08 g L⁻¹ h⁻¹) (Table S4). PPKs with broad substrate scope remain important tools (Matsumoto et al. 2026; Meng et al. 2025), but they and acid phosphatases present practical drawbacks: APs require low pH, forcing enzyme removal and pH adjustment before the downstream steps (Meng et al. 2025), and both AP‐ and PPK‐based cascades show substrate preferences that often lead to uneven product distributions (Matsumoto et al. 2026; Meng et al. 2025).

#### 3.3.5 Extension to Noncanonical Nucleosides and Nucleoside Analogs

Table S5 lists nucleoside analogs shown in previous studies to be substrates for the NK types used here. The noncanonical nucleoside pseudouridine (Ψ) is also phosphorylated by *Ec*UK. The NMPKs used (*Sc*URA6, *Sc*GMPK) exhibit a substrate scope that includes various NMP analogs. *Sc*URA6 in particular uses ΨMP and derivatives thereof with modification in the sugar (e.g., d‐arabinose, d‐xylose) and nucleobase (e.g., 2‐thio‐uracil; 6‐amino‐uracil) parts of the molecule (Supporting Information Table S5). *Ec*AcK offers an extremely relaxed specificity for NDP phosphorylation (Supporting Information Table S5). Demonstrating the function of our NK‐NMPK‐AcK cascades for NTP production from all these nucleosides was beyond the scope of the current study. However, we show clean conversion of 5 additional NMP substrates (Supporting Information Table S5) into the corresponding NTPs, with conversions in the range 67%–90% and product concentrations in the range of 7–41 mM (Supporting Information Figure S15–16). The results support the broad applicability of the NMPK‐AcK cascade to NTP synthesis from NMP analog substrates. By plausible extension based on NK substrate specificity, the full NK‐NMPK‐AcK cascade holds great promise as a synthetic tool with flexible use across a range of nucleoside analogs.

## 4 Conclusion

We demonstrated that *Sc*URA6 is a highly promiscuous nucleoside monophosphate kinase that efficiently phosphorylates AMP, CMP, and UMP, enabling cascades for ATP, CTP, and UTP synthesis. Substitution with *Sc*GMPK further extended the enzymatic platform to GTP production. Guided by kinetic and thermodynamic considerations, the cascades were rationally designed to ensure efficient intermediate transfer between enzymatic steps, thereby preventing intermediate accumulation. As a result, the reactions proceeded with high efficiency and robustness, facilitating simplified downstream processing and affording isolated NTPs up to 89% isolated yield and 99% purity. A modified format, wherein the NTP product serves as the phosphate shuttle, achieved conversions up to 98%. Owing to its modular, enzyme‐efficient design in combination with the promiscuity of the enzymes utilized (Supporting Information Table S5), this approach should readily extend to the synthesis of modified nucleotides. The cascades can provide a sustainable, cost‐effective framework for applications in nucleotide therapeutics and synthetic biology and, more broadly, a blueprint for future multi‐enzyme pathways targeting structurally complex nucleotide derivatives.

## Author Contributions

Martin Pfeiffer and Bernd Nidetzky designed the study. Oliver T. Damm and Martin Pfeiffer performed the experiments and analyzed the data. Johannes Zöhrer synthesized ΨTP and its analogs. Oliver T. Damm wrote the article, while Martin Pfeiffer and Bernd Nidetzky edited the article. Bernd Nidetzky acquired funding, and both Bernd Nidetzky and Martin Pfeiffer supervised the study.

## Conflicts of Interest

The authors declare no conflicts of interest.

## Supporting information

Supporting File 1

Supporting File 2

## Data Availability

The data that supports the findings of this study are available in the supporting material of this article.
