# Supplementary material for: A Modular Nucleoside Kinase Cascade for the Synthesis of Ribonucleoside Triphosphates
Source: Biotechnol Bioeng. 2026 May 24;123(8):2180–91. doi: 10.1002/bit.70247 (PMC13397275; doi:10.1002/bit.70247)
Supplement: Supplementary file 1 — Supporting File 1 [file BIT-123-2180-s001.pdf]

Supplementary ^{1}H and ^{13}C NMR spectra of the synthesized compounds.

Figure N1. ^{1}H NMR data of the synthesized ATP. 2

Figure N2. ^{13}C NMR data of the synthesized ATP. 3

Figure N3. ^{1}H NMR data of the synthesized CTP. 4

Figure N4. ^{13}C NMR data of the synthesized CTP. 5

Figure N5. ^{1}H NMR data of the synthesized GTP. 6

Figure N6. ^{13}C NMR data of the synthesized GTP. 7

Figure N7. ^{1}H NMR data of the synthesized UTP. 8

Figure N8. ^{13}C NMR data of the synthesized UTP. 9

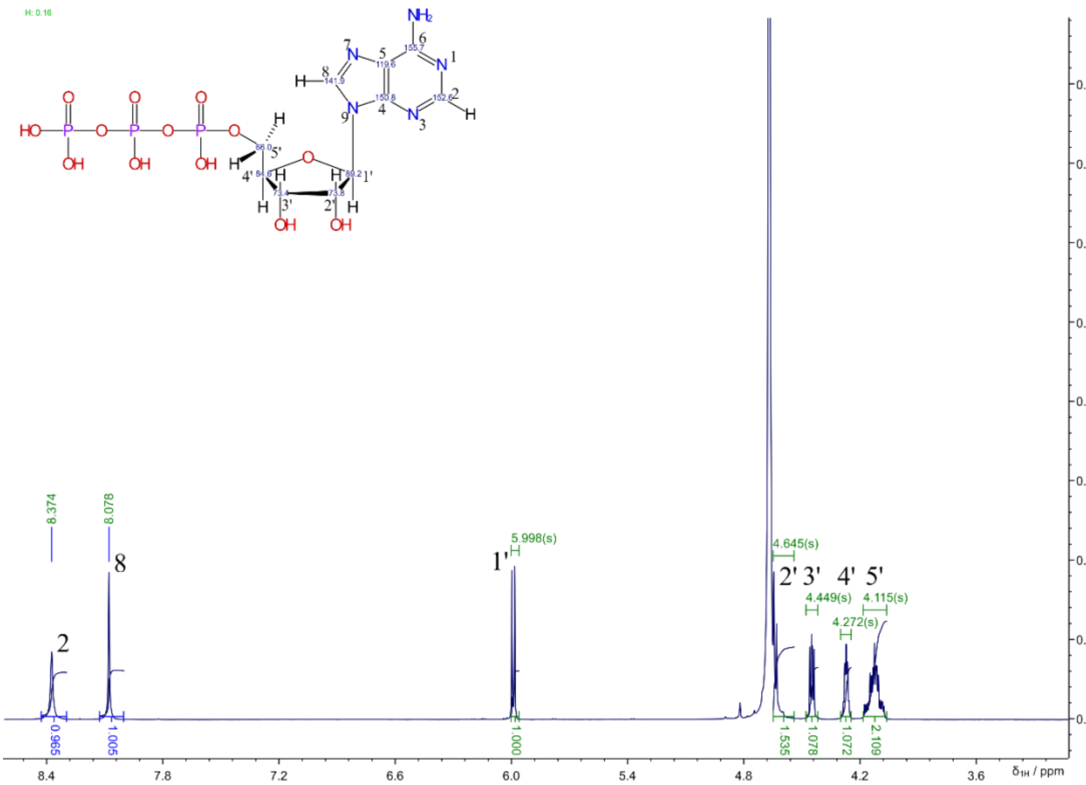

Figure N1. ^{1}H NMR data of the synthesized ATP. ^{1}H NMR (400 MHz, D_2O) δ: 8.37 (s, 1H, 2), 8.08 (s, 1H, 8), 6.00 (s, 1H, 1’), 4.64 (s, 1H, 2’), 4.45 (s, 1H, 3’), 4.27 (s, 1H, 4’), 4.12 (s, 2H, 5’).

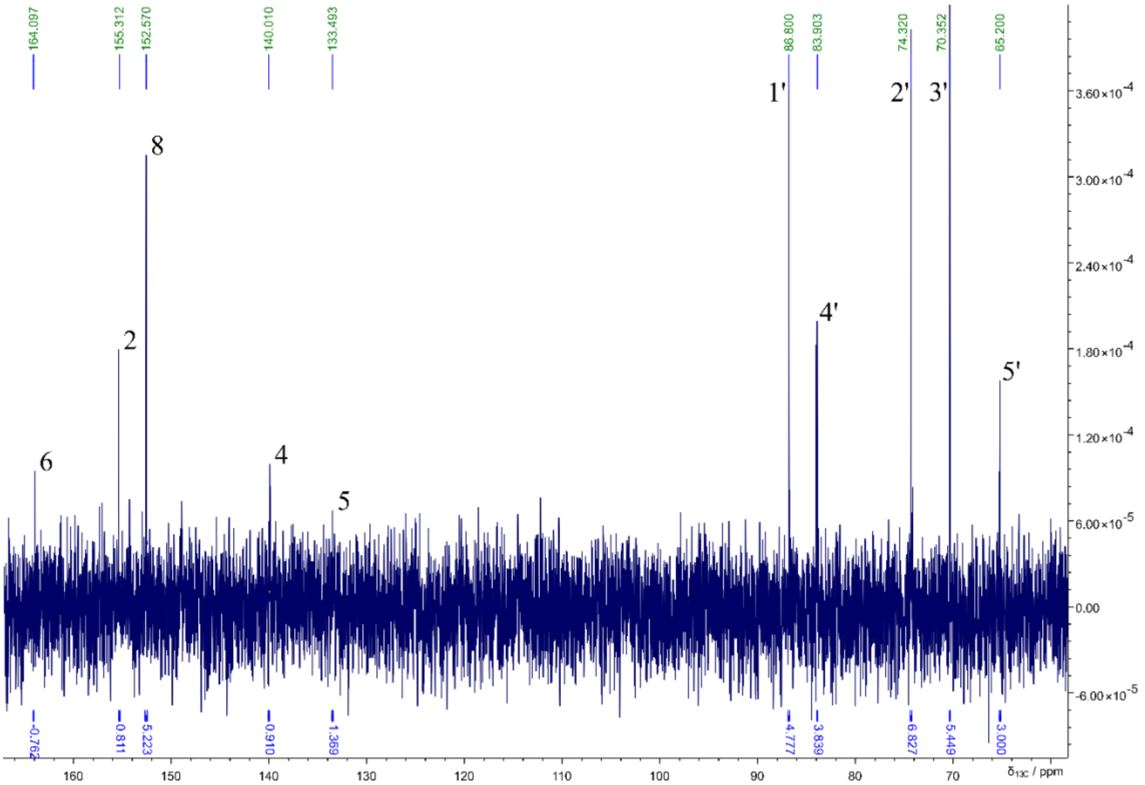

Figure N2. ^{13}C NMR data of the synthesized ATP. ^{13}C NMR (101 MHz, D_2O) δ: 163.40 - 164.43 (s, 0C, 6), 155.09 - 155.83 (s, 0C, 2), 152.57 (s, 1C, 8), 139.79 - 140.23 (s, 1C, 4), 133.02 - 134.05 (s, 0C, 5), 86.80 (s, 1C, 1’), 83.46 - 84.27 (s, 1C, 4’), 74.32 (s, 1C, 2’), 70.35 (s, 1C, 3’), 64.87 - 65.50 (s, 0C, 5’).

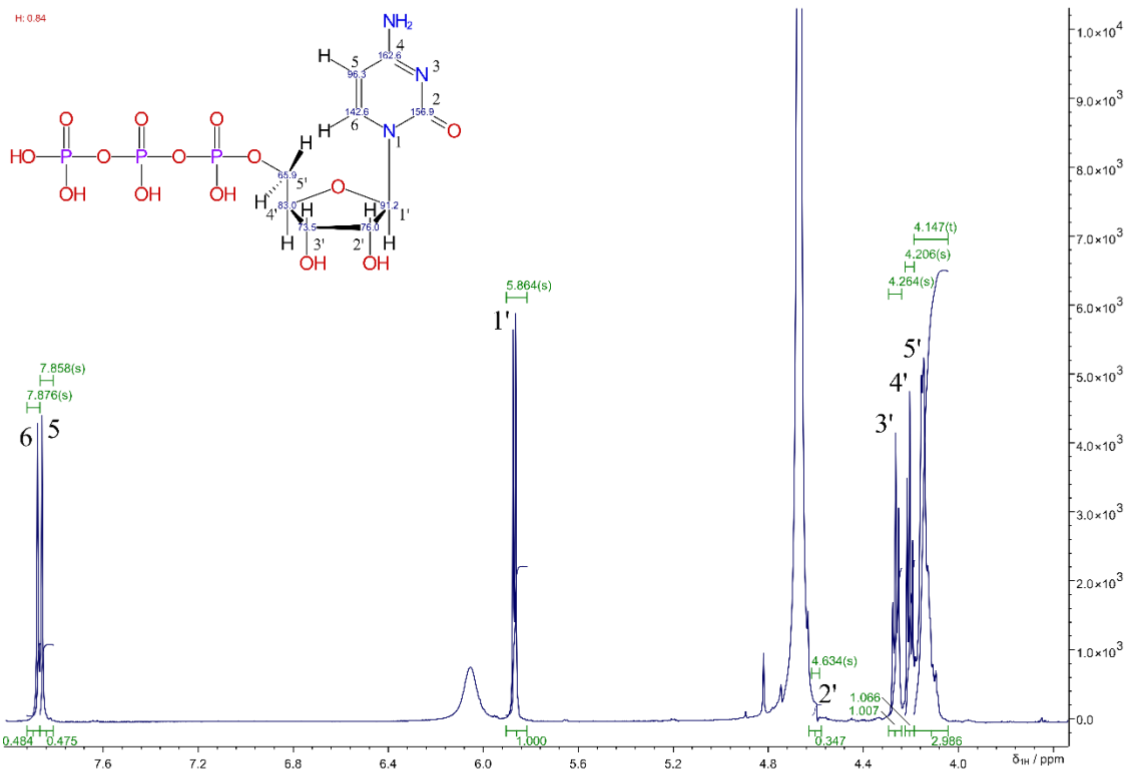

Figure N3. ^{1}H NMR data of the synthesized CTP. ^{1}H NMR (400 MHz, D_2O) δ: 7.88 (s, 1H, 6), 7.86 (s, 1H, 5), 5.86 (s, 1H, 1'), 4.63 (s, 1H, 2'), 4.26 (s, 1H. 3'), 4.20 (s, 1H, 4'), 4.15 (s, 2H, 5').

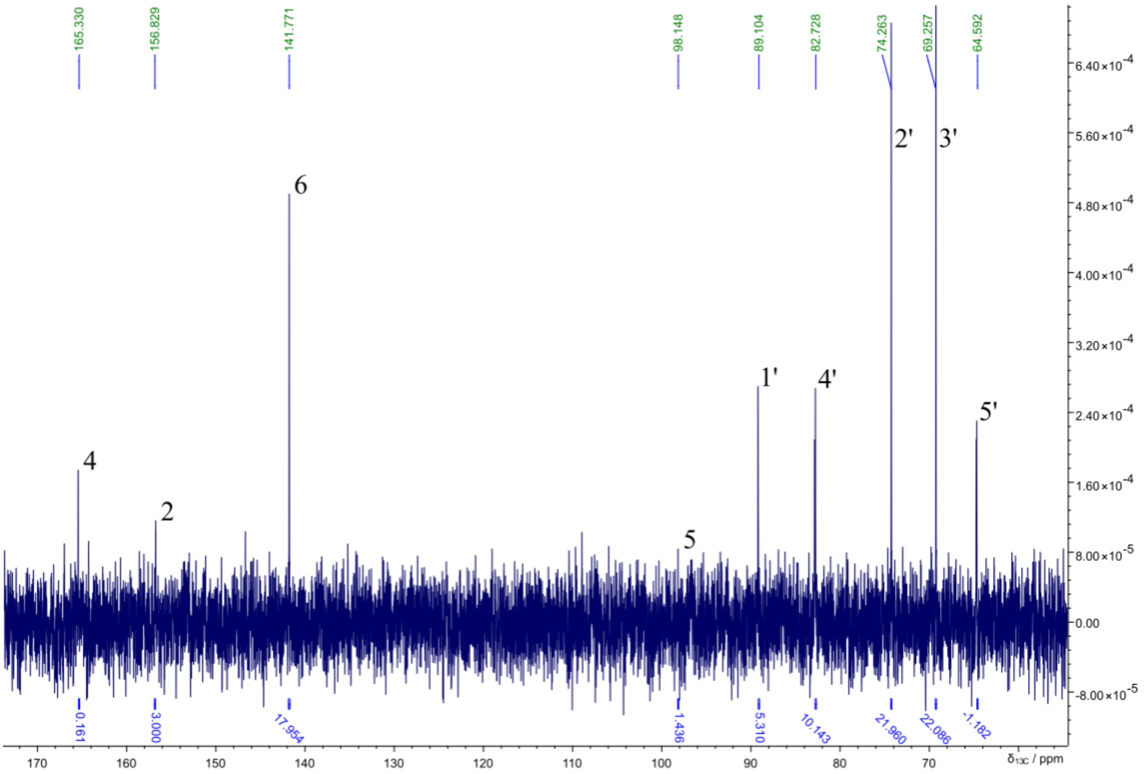

Figure N4. ^{13}C NMR data of the synthesized CTP. ^{13}C NMR (101 MHz, D_2O) δ: 165.10 - 165.86 (s, 8C, 4), 156.31 - 157.15 (s, 3C, 2), 141.77 (s, 9C, 6), 98.02 - 98.48 (s, 0C, 5), 89.08 - 89.46 (s, 9C, 1'), 82.51 - 83.20 (s, 10C, 4'), 74.26 (s, 11C, 2'), 69.26 (s, 12C, 3'), 64.48 - 65.01 (s, 6C, 5').

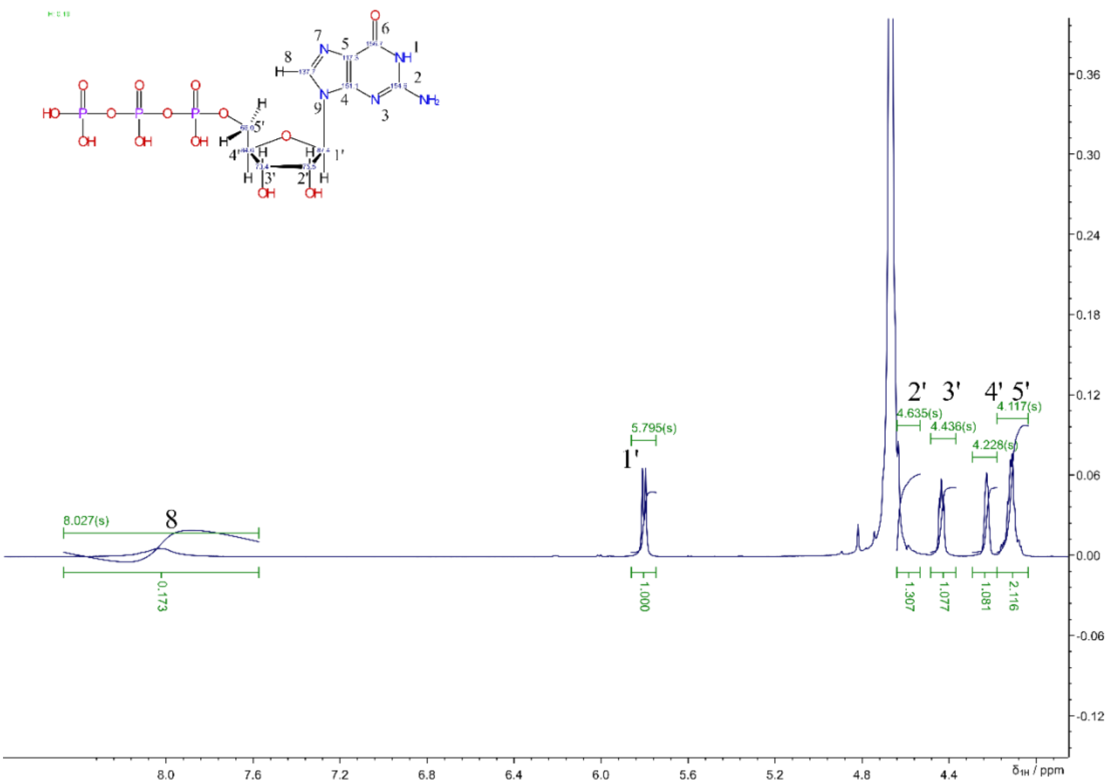

Figure N5. ^{1}H NMR data of the synthesized GTP. ^{1}H NMR (400 MHz, D_2O) δ: 8.03 (s, 1H, 8), 5.80 (s, 1H, 1'), 4.63 (s, 1H, 2'), 4.44 (s, 1H, 3'), 4.23 (s, 1H, 4'), 4.12 (s, 2H, 5').

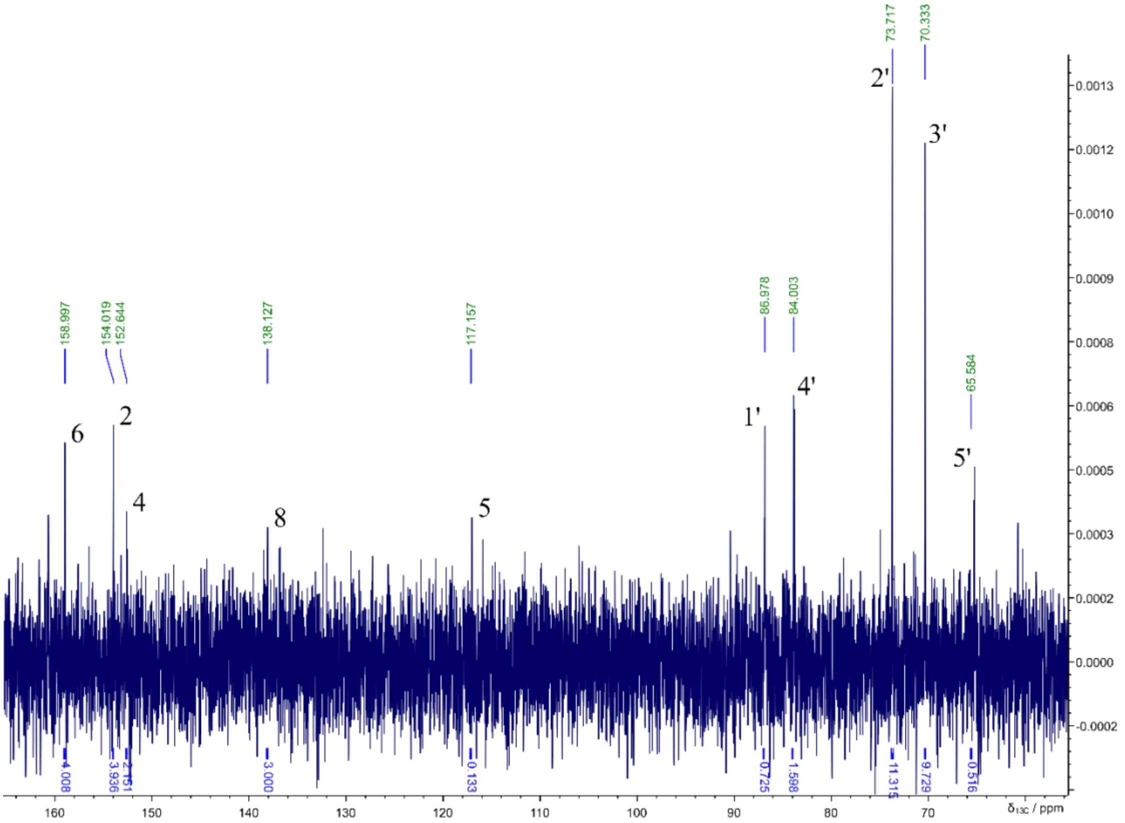

Figure N6. ^{13}C NMR data of the synthesized GTP. ^{13}C NMR (101 MHz, D_2O) δ: 158.83 - 159.18 (s, 3C, 6), 153.81 - 154.27 (s, 6C, 2), 152.31 - 152.85 (s, 4C, 4), 137.93 - 138.27 (s, 5C, 8), 116.99 - 117.10 (s, 0C, 5), 86.27 - 87.69 (s, 9C, 1’), 83.29 - 84.71 (s, 15C, 4’), 73.72 (s, 7C, 2’), 70.33 (s, 9C, 3’), 64.45 - 66.15 (s, 1C, 5’).

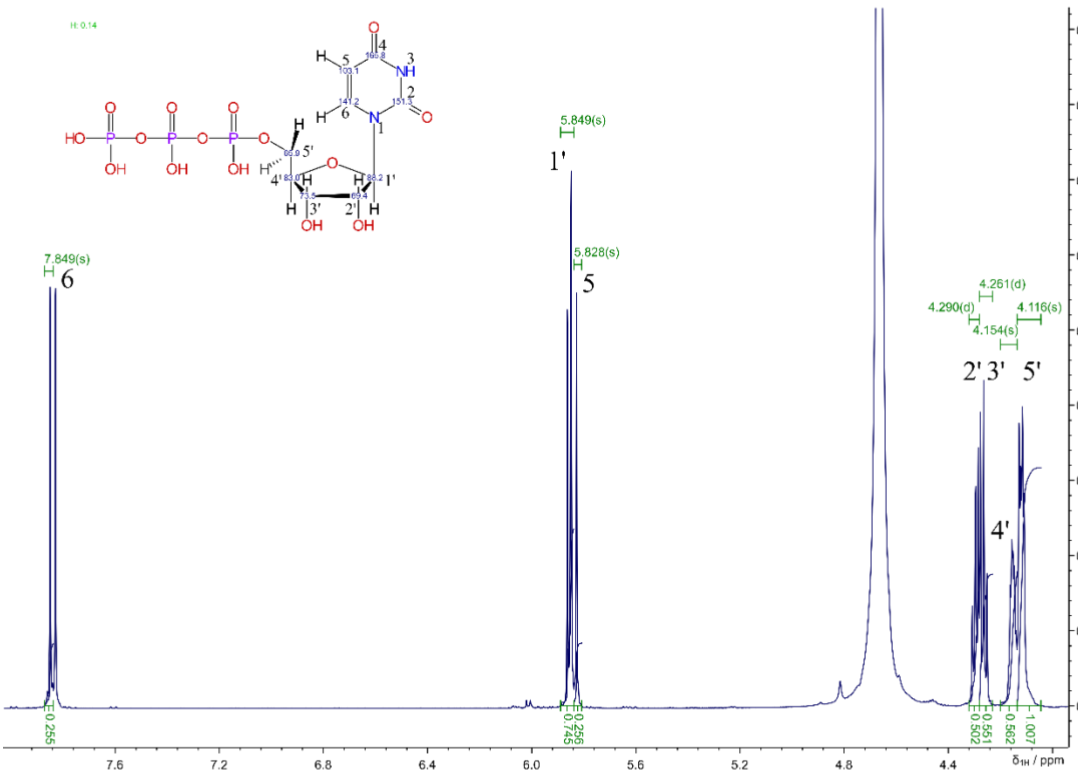

Figure N7. ^{1}H NMR data of the synthesized UTP. ^{1}H NMR of UTP (400 MHz, D_2O) δ: 7.85 (s, 1H, 6), 5.85 (s, 1H, 1'), 5.83 (s, 1H, 5), 4.29 (d, J: 4.4, 1H, 2'), 4.26 (d, J: 5.1, 1H, 3'), 4.15 (s, 1H, 4'), 4.12 (s, 2H, 5').

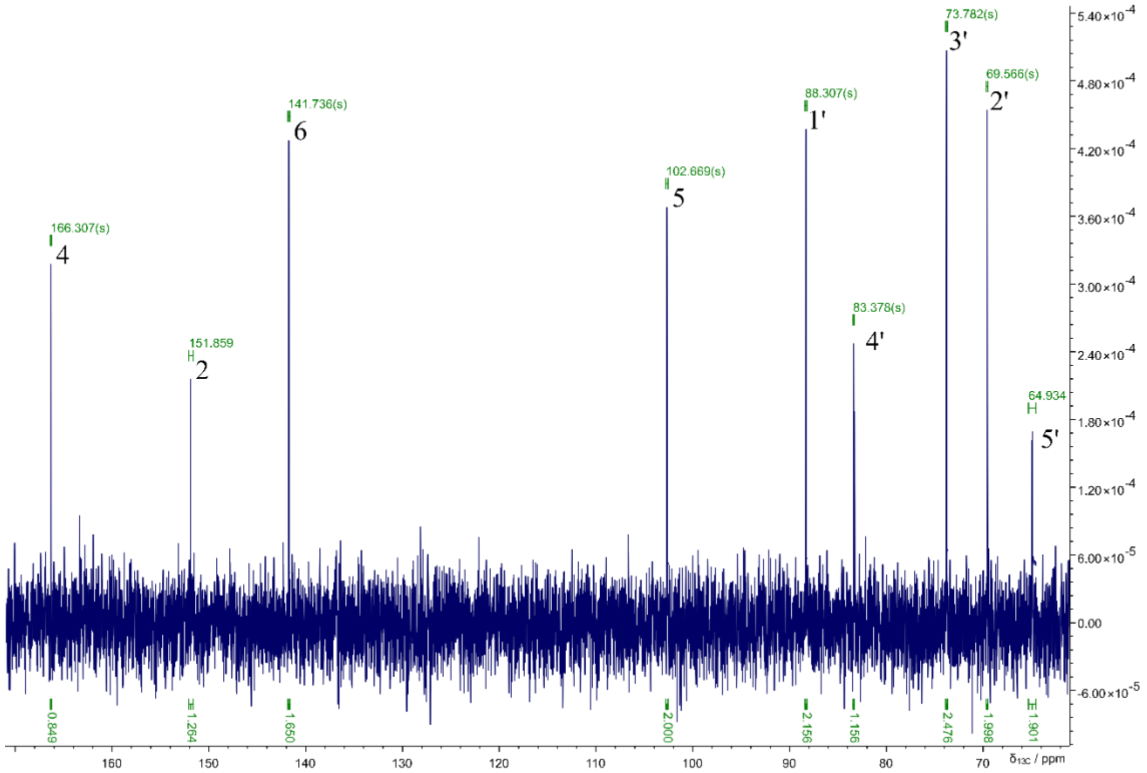

Figure N8. ^{13}C NMR data of the synthesized UTP. ^{13}C NMR of UTP (101 MHz, D_2O) δ: 166.31 (s, 1C, 4), 151.09 - 153.10 (s, 1C, 2), 141.74 (s, 2C, 6), 102.67 (s, 2C, 5), 88.31 (s, 2C, 1’), 83.38 (s, 1C, 4’), 73.78 (s, 2C. 3’), 69.57 (s, 2C, 2’), 64.88 - 65.13 (s, 2C, 5’).
