# Supplementary material for: A Modular Nucleoside Kinase Cascade for the Synthesis of Ribonucleoside Triphosphates
Source: Biotechnol Bioeng. 2026 May 24;123(8):2180–91. doi: 10.1002/bit.70247 (PMC13397275; doi:10.1002/bit.70247)
Supplement: Supplementary file 2 — Supporting File 2 [file BIT-123-2180-s002.pdf]

Supporting Information

A Modular Nucleoside Kinase Cascade for the Synthesis of Ribonucleoside Triphosphates

Oliver T. Damm[1], Martin Pfeiffer[1], Johannes Zöhrer[1], Bernd Nidetzky[1,2,*]

[1] Institute of Biotechnology and Biochemical Engineering, Graz University of Technology, NAWI Graz, Petersgasse 12, A-8010 Graz, Austria

[2] Austrian Centre of Industrial Biotechnology (acib), Krenngasse 37, A-8010 Graz, Austria

* Corresponding author (B. N.; email: bernd.nidetzky@tugraz.at)

Table of Contents

1. Table of figures 3

2. Table of tables 3

3. Supporting Methods 4

3.1. Cloning and strain creation 4

3.2. Enzyme expression and purification 4

3.3. AcP synthesis 5

3.4. Analysis 5

3.4.1. Reversed-phase HPLC (RP-HPLC) 5

3.4.2. Spectrophotometric concentration analysis 6

3.4.3. Nuclear magnetic resonance spectroscopy 6

Gene and plasmid sequences 22

3.5. pET-15b(+) 22

3.5.1. vector sequence 22

3.5.2. *Ec*UK 23

3.5.3. *Sc*URA6 23

3.5.4. *Ec*AcK 24

3.6. pET-28a(+) 24

3.6.1. vector sequence 24

3.6.2. *Ea*GK, Seq. 1 26

3.6.3. *Sc*AK, Seq. 2 27

3.6.4. *Sc*GMPK, Seq. 3 28

5. Supporting References 28

1. Table of figures

Figure S1. SDS-PAGE of purified enzymes used in this study. 7
Figure S2. NK activity characterization. 8
Figure S3. NMPK activity characterization. 9
Figure S4. *Ec*AcK activity characterization. 10
Figure S5. Substrate binding of purine and pyrimidine nucleotides in the active sites of *Sc*URA6 (pink) and *Dd*URA6 (cyan). 11
Figure S6. Active-site interactions of the guanine moiety in the crystal structure (PDB ID 1EX7) of *Sc*GMPK (yellow) bound to GMP (white). 12
Figure S7. Enzyme loading optimization in the C cascade. 13
Figure 8. $MgCl_2$ optimization in the 100 mM C cascade. 14
Figure S9. C cascade intensification from 10 to 100 mM (final iteration). 15
Figure S10. Comparison of C (squares) and U (circles) cascades at a 50 mM and b 100 mM substrate. 16
Figure S11. Conversion of G to GTP measured by RP-HPLC 16
Figure S12. G cascade intensification at a 10 mM and b 25 mM. 16
Figure S13. AEX chromatogram for ATP purification. 17
Figure S14. Auto-regeneration cascades for NTP production. 18
Figure S15. Scheme of the enzymatic phosphorylation cascade used to synthesize ΨTP and its analogues. Y_R, reaction yield in %. 19
Figure S16. Time course of the enzymatic phosphorylation of ΨMP to and its analogues. 19

2. Table of tables

Table S1. Typical retention time (t_R) of the analyzed nucleosides and nucleotides. 20
Table S2. Primers used in this study. 20
Table S3. Summary of cascade intensification at 1 mL scale. 20
Table S4. Summary of key parameters for NTP production from nucleosides. 21
Table S5. Reported additional substrates for enzymes used in this study (excluding the substrates applied here). 21

3. Supporting Methods

3.1. Cloning and strain creation

Genes encoding *Ea*GK (Seq. 1), *Sc*AK (Seq. 2), and *Sc*GMPK (Seq. 3) were synthesized (GenScript, Rijswijk, Netherlands) with 5′ and 3′ overhangs complementary to the pET-28a(+) vector.
5′ overhang: (CTAGAAATAATTTTGTTTAACTTTAAGAAGGAGATATACC)
3′ overhang: (CACCACCACCACCACCACTGAGATC).
The pET-28a(+) vector was linearized by PCR using pET-28a(+)_fwd and pET-28a(+)_rev primers (Table S2) and Q5® High-Fidelity DNA Polymerase (New England Biolabs GmbH, Ipswich, MA, USA). PCR conditions: initial denaturation at 98 °C for 30 s; 33 cycles of 98 °C for 5 s, 50 °C for 15 s, and 72 °C for 180 s; final elongation at 72 °C for 60 s. The product was treated with DpnI and purified using the Wizard SV Gel and PCR Clean-Up System (Promega, Madison, WI, USA).

Purified vector and inserts were assembled using NEBuilder HiFi DNA Assembly Master Mix (New England Biolabs) for 45 min at 50 °C and transformed into *E. coli* NEB 10-beta. Transformants were selected on LB agar (50 µg mL^{-1} kanamycin). Plasmids were isolated (Wizard Plus SV Minipreps, Promega) and verified by Sanger sequencing (Microsynth, Balgach, Switzerland) using T7 promoter and terminator primers. Correct constructs were introduced into *E. coli* NiCo21 (DE3) for expression and glycerol stock preparation.

3.2. Enzyme expression and purification

Proteins were expressed in 1 L baffled shake flasks containing 250 mL TB medium supplemented with 50 µg mL^{-1} kanamycin (*Ea*GK, *Sc*AK, and *Sc*GMPK) or 100 µg mL^{-1} ampicillin (*Ec*UK, *Sc*URA6, and *Ec*AcK). Main cultures were inoculated from overnight precultures to an OD_{600} = 0.1, grown at 37 °C and 120 rpm to an OD_{600} of 0.8 – 1.0, induced with 0.4 mM IPTG, and incubated for 20 h at 20 °C. Cells were harvested at 2000 × g (Sorvall® Evolution™ RC Superspeed Centrifuge, Thermo Fisher Scientific, Waltham, MA, USA).

Pellets were resuspended in His-tag binding buffer (20 mM HEPES, 0.5 M NaCl, 20 mM imidazole, pH 7.5) and lysed by addition of lysozyme and sonication (Vibra Cell Processor VCX130, Sonics & Materials Inc., Newton, CT, USA) for 8 min (2 s on / 4 s off, 40 % amplitude). Debris was removed at 20,000 × g to obtain the clarified cell-free extract (CFE).

His_6-tagged enzymes were purified by Ni-NTA affinity chromatography. A 40 mL aliquot of lysate was loaded onto a 5 mL HisTrap™ HP column (Cytiva, Marlborough, MA, USA) on an ÄKTAprime Plus system (Cytiva), equilibrated with His-tag binding buffer, and eluted at 10 °C with a linear gradient (0 – 100%) of elution buffer (20 mM HEPES, 0.5 M NaCl, 300 mM imidazole, pH 7.5) over 120 min.

Fractions containing the target enzyme were pooled and concentrated using Vivaspin™ Turbo 15 RC ultrafiltration units (10 kDa or 30 kDa cutoff, Sartorius, Göttingen, Germany) at 3095 × g and 4 °C, with simultaneous buffer exchange into protein storage buffer (50 mM HEPES, 1 mM $MgCl_2$, 125 mM NaCl, 5 % glycerol, pH 7.5). Purity was confirmed by SDS-PAGE (Figure S1). Purified enzymes were stored at -20 °C.

3.3. AcP synthesis

Acetyl phosphate (AcP) was prepared following Tasnádi et al. [1] Briefly, 1.7 mL aqueous H_3PO_4 was combined with 15 mL ethyl acetate (EtOAc) and cooled to 4 °C with stirring. 7.1 mL acetic anhydride (precooled to 4 °C) was added dropwise. The mixture was stirred at 4 °C for 5.5 h.

The reaction was quenched by adding 10 mL water, 6.3 g ice, and 2.1 g $NaHCO_3$. The aqueous phase was washed three times with 25 mL ice-cold EtOAc. The pH was adjusted to 7.0 using 10 M NaOH, followed by an additional wash with 6 mL EtOAc. Residual solvent was removed by rotary evaporation (Laborota 4000 efficient; 30 °C, 30 mbar, 150 rpm).

3.4. Analysis

3.4.1. Reversed-phase HPLC (RP-HPLC)

Samples (60 µL) were quenched by 1:2 dilution with methanol, centrifuged for 30 min at 21300 × *g* (Eppendorf 5425R, Hamburg, Germany), and analyzed by loading 10 µL on a Kinetex 5 µm C18 (100 Å) column (Phenomenex Ltd., Aschaffenburg, Germany) using a Shimadzu LC-20 HPLC system (Shimadzu Europe GmbH, Duisburg, Germany) with UV detection at 262 nm. Separation was performed at 40 °C and 1.5 mL min^{-1} using 84 % tetrabutylammonium bromide (TBAB; 40 mM) buffer (20 mM potassium phosphate, pH 5.9) and 16 % acetonitrile. Elution was monitored at 262 nm. Data acquisition and analysis were carried out using LabSolutions CS (version 5.110). Typical retention times are listed in Table S1.

3.4.2. Spectrophotometric concentration analysis

Protein concentrations were determined at 280 nm (Nanophotometer N50, Implen, Munich, Germany) from 1 µL samples using molar extinction coefficients from ExPASy ProtParam and the respective molecular weights. Concentrations are reported in mg mL^{-1}.

Molar extinction coefficients for ATP and GTP under reaction conditions were determined experimentally. Stock solutions (50 mM) containing all reaction compounds except enzymes (200 mM AcP, 50 mM $MgCl_2$ and 50 mM TAPS) were prepared and diluted to 2 mM. Serial dilutions to 0.2 mM were measured in triplicate at 260 nm (1 µL). Linear regression of absorbance versus the concentration yielded the molar extinction coefficients of 14.8 mM^{-1} cm^{-1} (ATP) and 11.5 mM^{-1} cm^{-1} (GTP).

For reactions employing A or G, substrate dissolution was monitored spectrophotometrically. Samples were centrifuged (5 min, 21300 × *g*), supernatants were diluted to 0.5 mM total nucleoside/nucleotide and measured at 260 nm. Total dissolved nucleoside/nucleotide concentrations were calculated accordingly and combined with RP-HPLC speciation to determine conversions.

3.4.3. Nuclear magnetic resonance spectroscopy

Freeze-dried products were dissolved in D_2O prior to analysis. NMR spectra were recorded on a JEOL JNM-ECZL 400 MHz NMR spectrometer (^{1}H: 399.78 MHz, ^{13}C: 100.53 MHz, ^{31}P: 161.83 MHz). Chemical shifts (δ) are reported in ppm; coupling constants (*J*) in Hz. ^{1}H chemical shifts were referenced to the residual D_2O signal at $\delta = 4.79$ ppm. Signal multiplicities are abbreviated as s (singlet) and d (doublet).

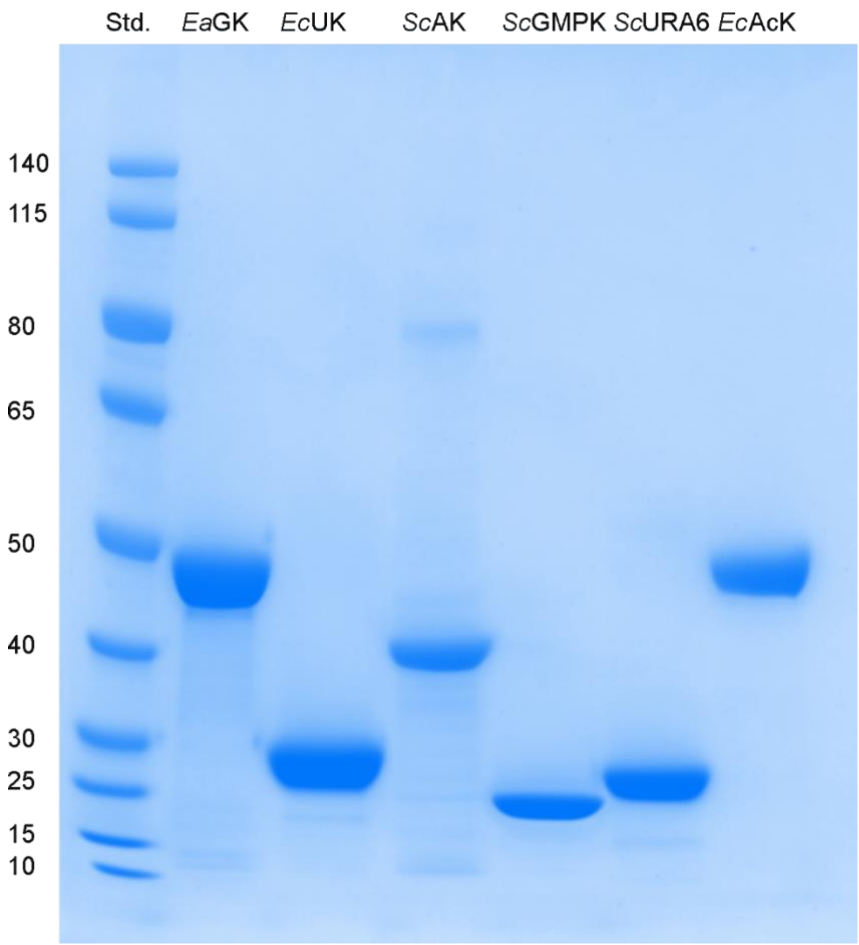

Figure S1. SDS-PAGE of purified enzymes used in this study. Proteins were separated at 210 V for 45 min and stained with Coomassie Brilliant Blue. Lanes are annotated with enzyme identities. Std, PageRuler PLUS Prestained Protein Ladder (10 – 140 kDa).

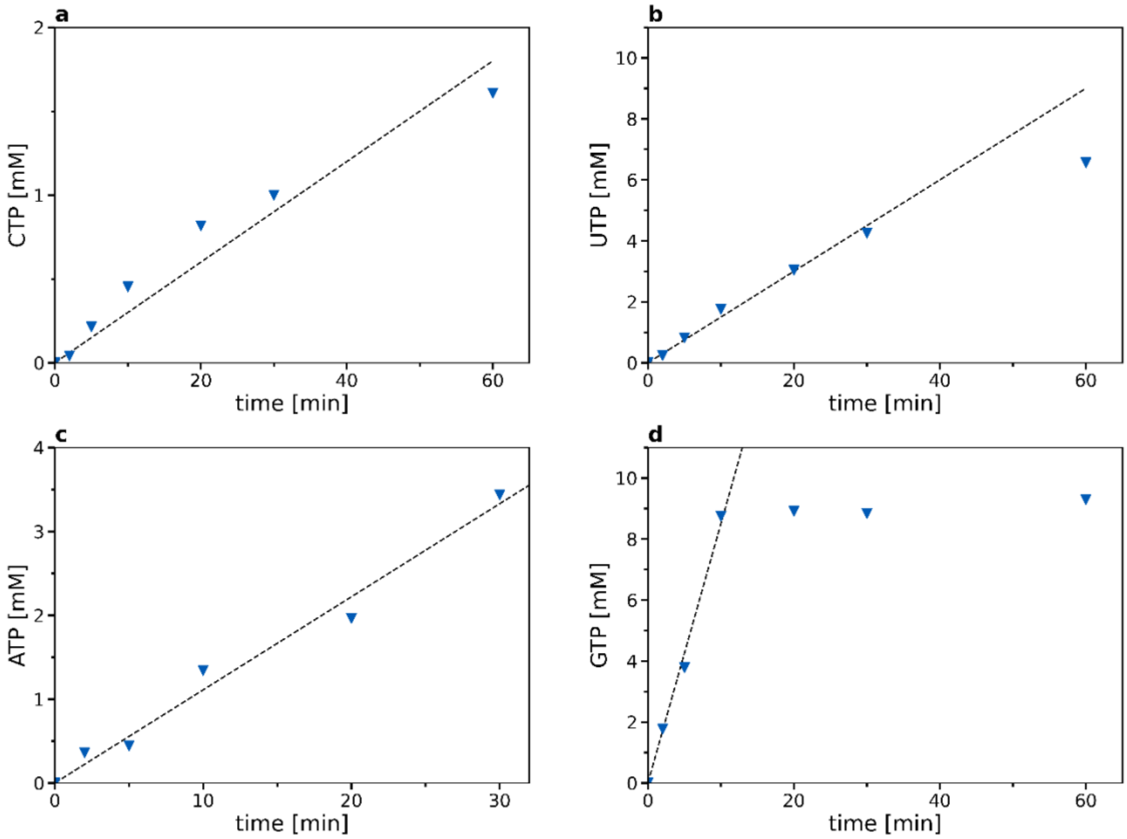

Figure S2. NK activity characterization. Activity of *Ec*UK on **a** C and **b** U; *Sc*AK on **c** A; *Ea*GK on **d** G. Conditions: 1.0 mL, 50 mM TAPS (pH 8.0), 30 °C, 300 rpm; 10 mM nucleoside, 0.5 mM ATP (for G and U) or GTP (for A and C), 40 mM AcP, 10 mM $MgCl_2$. Enzyme loadings: **a** 0.3 U mL^{-1} *Ec*UK, 33 U mL^{-1} *Sc*URA6, 121 U mL^{-1} *Ec*AcK; **b** 1.4 U mL^{-1} *Ec*UK, 32 U mL^{-1} *Sc*URA6, 447 U mL^{-1} *Ec*AcK; **c** 0.1 U mL^{-1} *Sc*AK, 82 U mL^{-1} *Sc*URA6, 413 U mL^{-1} *Ec*AcK; **d** 1.6 U mL^{-1} *Ea*GK, 32 U mL^{-1} *Sc*GMPK, 696 U mL^{-1} *Ec*AcK. NTP [mM], blue inverted triangles; linear regression [mM], black dashed line.

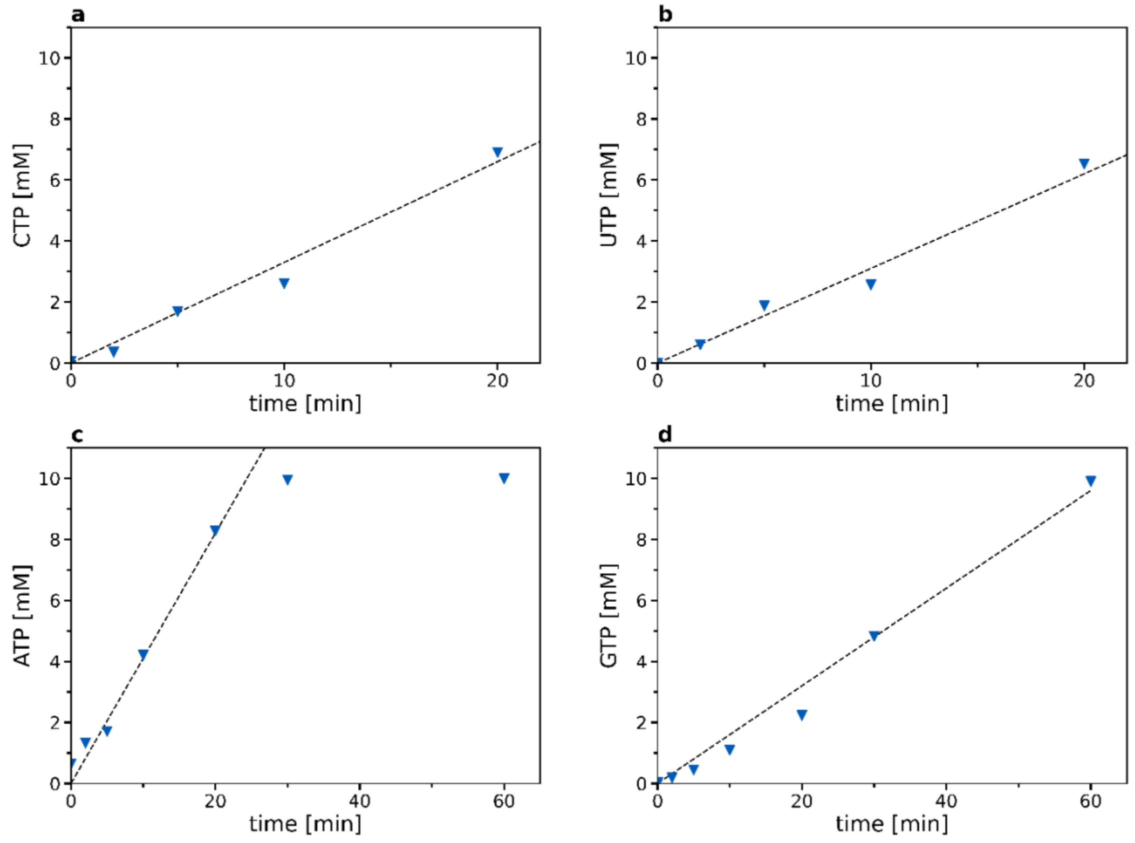

Figure S3. NMPK activity characterization. Reactions of *Sc*URA6 on **a** CMP, **b** UMP, and **c** AMP, and of *Sc*GMPK on **d** GMP. Conditions: 1.0 mL, 50 mM TAPS (pH 8.0), 30 °C, 300 rpm; 10 mM NMP, 0.5 mM ATP (for G and U) or GTP (for A and C), 40 mM AcP, 10 mM $MgCl_2$. Enzyme loadings: **a** 0.3 U mL^{-1} *ScURA6*, 121 U mL^{-1} *EcAcK*; **b** 0.3 U mL^{-1} *ScURA6*, 447 U mL^{-1} *EcAcK*; **c** 0.4 U mL^{-1} *ScURA6*, 413 U mL^{-1} *EcAcK*; **d** 0.2 U mL^{-1} *ScGMPK*, 696 U mL^{-1} *EcAcK*. NTP [mM], blue inverted triangles; linear regression [mM], black dashed line.

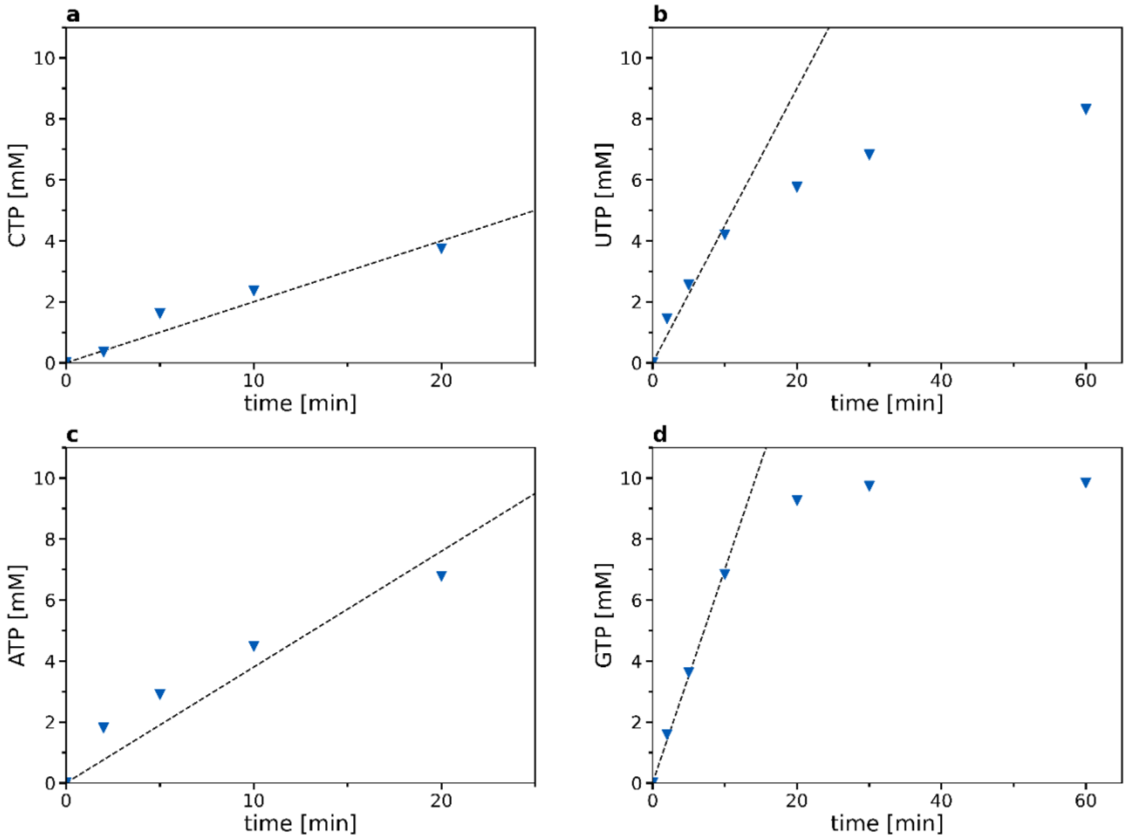

Figure S4. *Ec*AcK activity characterization. Reactions on **a** CDP, **b** UDP, **c** ADP, and **d** GDP. Conditions: 1.0 mL, 50 mM TAPS (pH 8.0), 30 °C, 300 rpm; 10 mM NDP, 15 mM AcP, 10 mM $MgCl_2$. **a** 0.3 U mL^{-1} *Ec*AcK; **b** 0.4 U mL^{-1} *Ec*AcK; **c** 0.8 U mL^{-1} *Ec*AcK; **d** 0.4 U mL^{-1} *Ec*AcK. NTP [mM], blue inverted triangles; linear regression [mM], black dashed line.

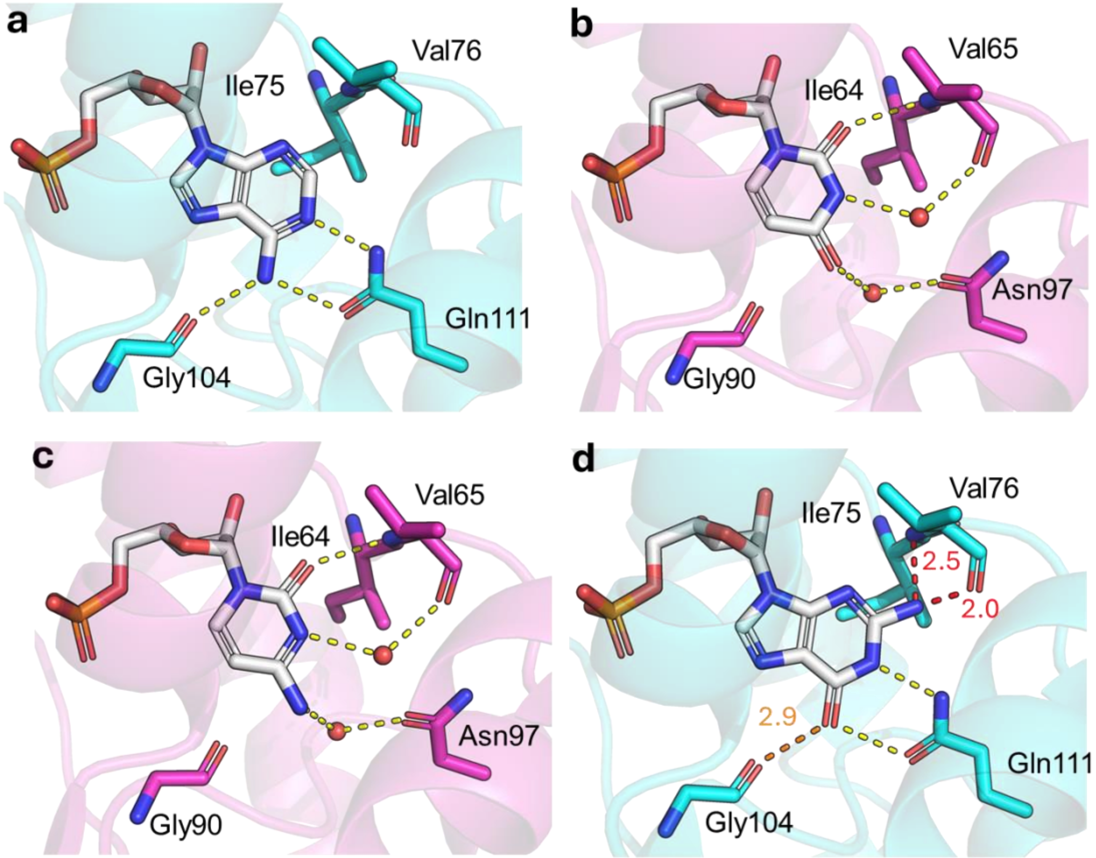

Figure S5. Substrate binding of purine and pyrimidine nucleotides in the active sites of *Sc*URA6 (pink) and *Dd*URA6 (cyan). **a** *Sc*URA6-ADP complex (PDB ID: 1UKY). **b** *Sc*URA6-uridine penta-phosphate complex (PDB ID: 1UKD). **c** *Sc*URA6–C penta-phosphate complex (C5 carbonyl replaced by amino group). **d** *Sc*URA6-GDP model generated by substituting ADP with GDP and rotating Gln111 of the ScURA6-ADP complex. Only NMP moieties are shown. Yellow dashed lines, polar interactions (≤3.3 Å); orange dashed lines, unfavorable interactions; red dashed lines, steric clashes. Distances in Å.

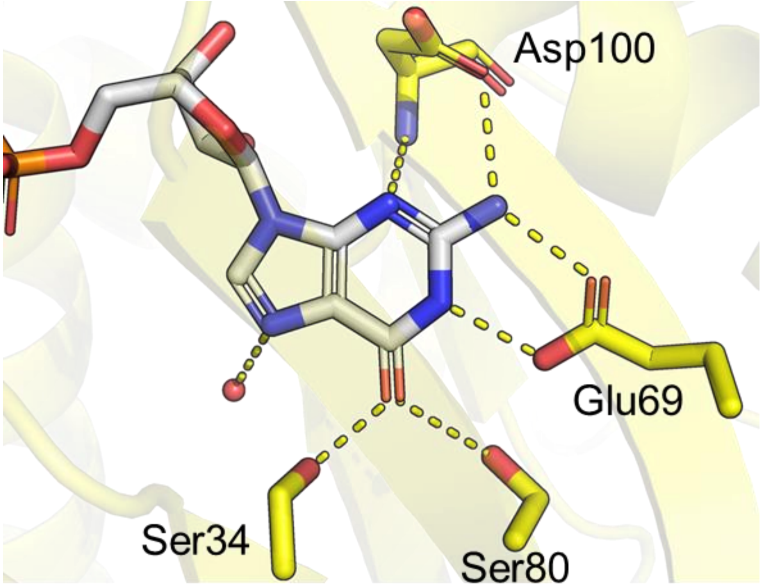

Figure S6. Active-site interactions of the guanine moiety in the crystal structure (PDB ID 1EX7) of *Sc*GMPK (yellow) bound to GMP (white). Yellow dashed lines represent polar interactions (≤ 3.3 Å).

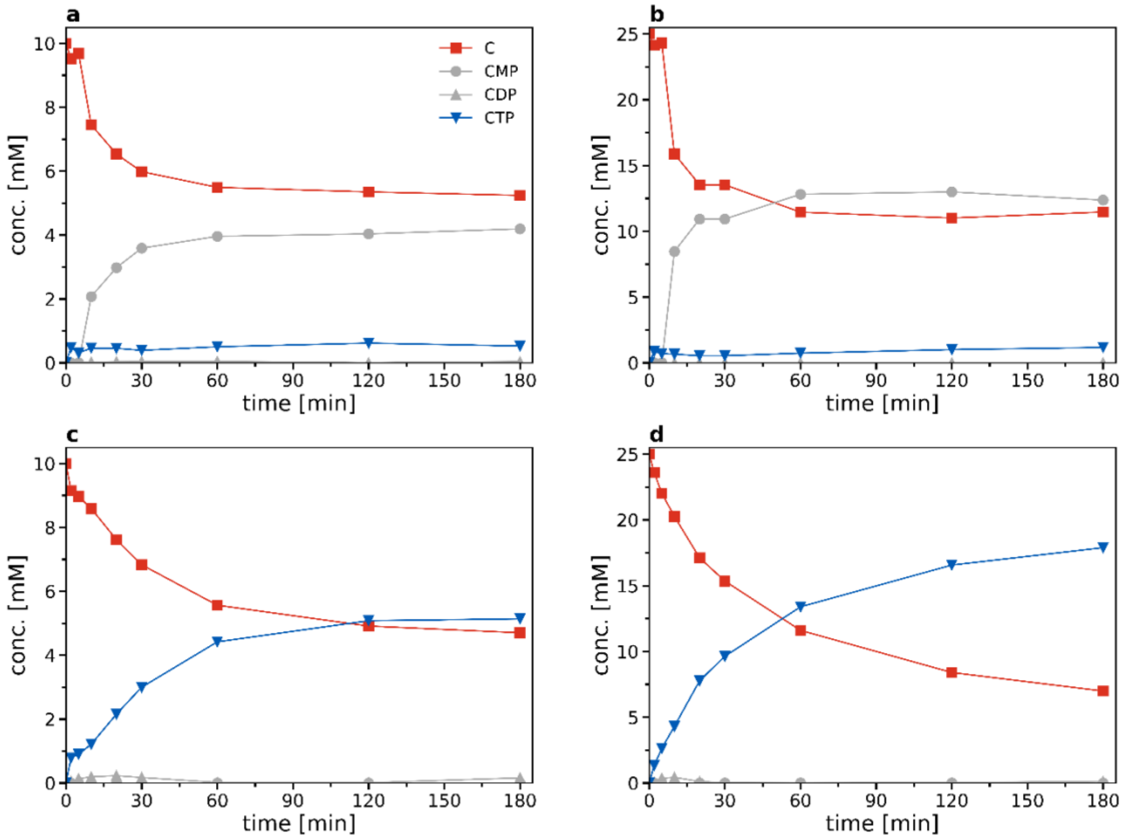

Figure S7. Enzyme loading optimization in the C cascade. C at 10 mM (**a**, **c**) and 25 mM (**b**, **d**). First iteration (**a**, **b**): limiting enzyme loadings targeting full conversion at 60 min; second iteration (**c**, **d**): *Sc*URA6 and *Ec*AcK at tenfold excess. Enzyme activities (10 mM): first, 0.17 U mL^{-1} *Ec*UK, 0.17 U mL^{-1} *Sc*URA6, 0.7 U mL^{-1} *Ec*AcK; second, 0.17 U mL^{-1} *Ec*UK, 1.7 U mL^{-1} *Sc*URA6, 7 U mL^{-1} *Ec*AcK. Enzyme activities (25 mM): first, 0.4 U mL^{-1} *Ec*UK, 0.4 U mL^{-1} *Sc*URA6, 1.7 U mL^{-1} *Ec*AcK; second, 0.4 U mL^{-1} *Ec*UK, 4 U mL^{-1} *Sc*URA6, 17 U mL^{-1} *Ec*AcK. Reaction components: 10 mM cascade, 0.5 mM GTP, 40 mM AcP, 10 mM $MgCl_2$; 25 mM cascade, 1.25 mM GTP, 100 mM AcP, 25 mM $MgCl_2$. Conditions: 1.0 mL, 50 mM TAPS (pH 8.0), 30 °C, 300 rpm.

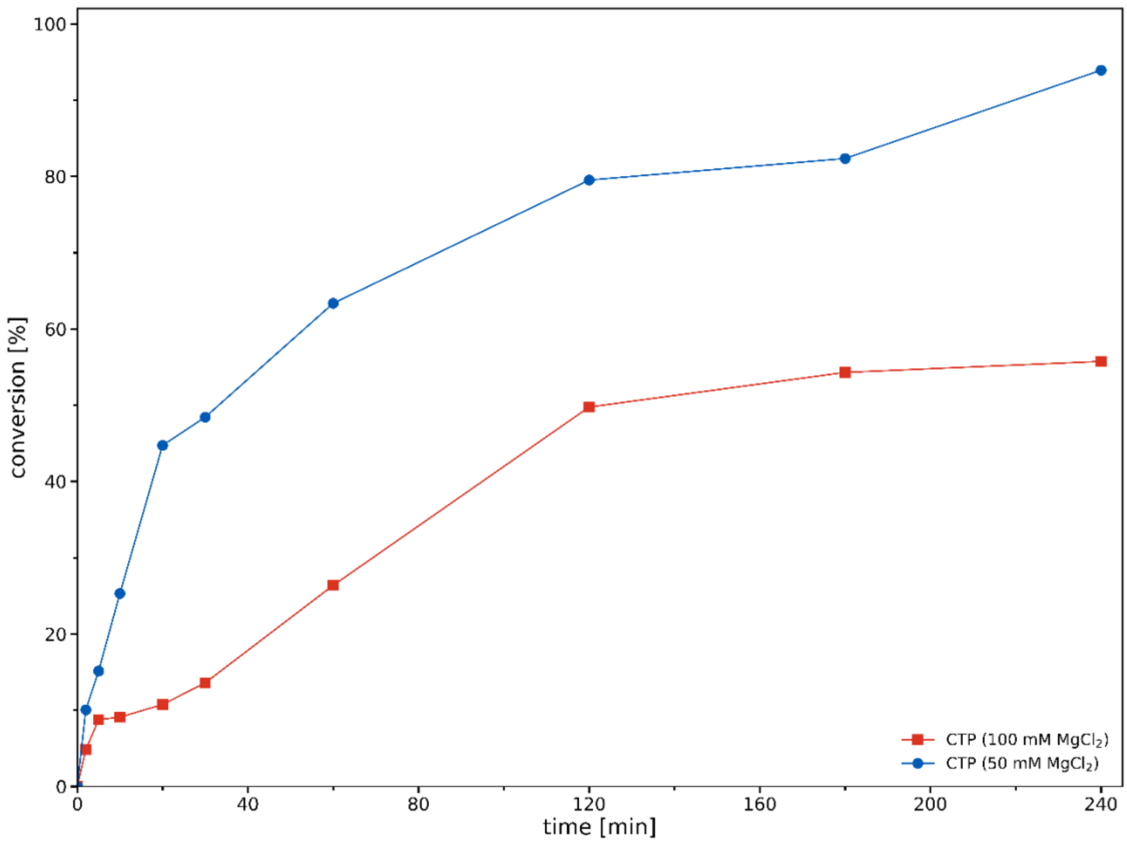

Figure S8. $MgCl_2$ optimization in the 100 mM C cascade. With 100 mM $MgCl_2$ (squares), conversion reached 54% at 240 min. With 50 mM $MgCl_2$ (circles, third iteration), conversion improved to 91% at 240 min and no $Mg(OH)_2$ precipitation was observed. Only CTP is shown; no intermediate accumulation was detected. Conditions: 3.3 U mL^{-1} *Ec*UK, 33.3 U mL^{-1} *Sc*URA6, 200 U mL^{-1} *Ec*AcK; 1.0 mL, 50 mM TAPS (pH 8.0), 30 °C, 300 rpm.

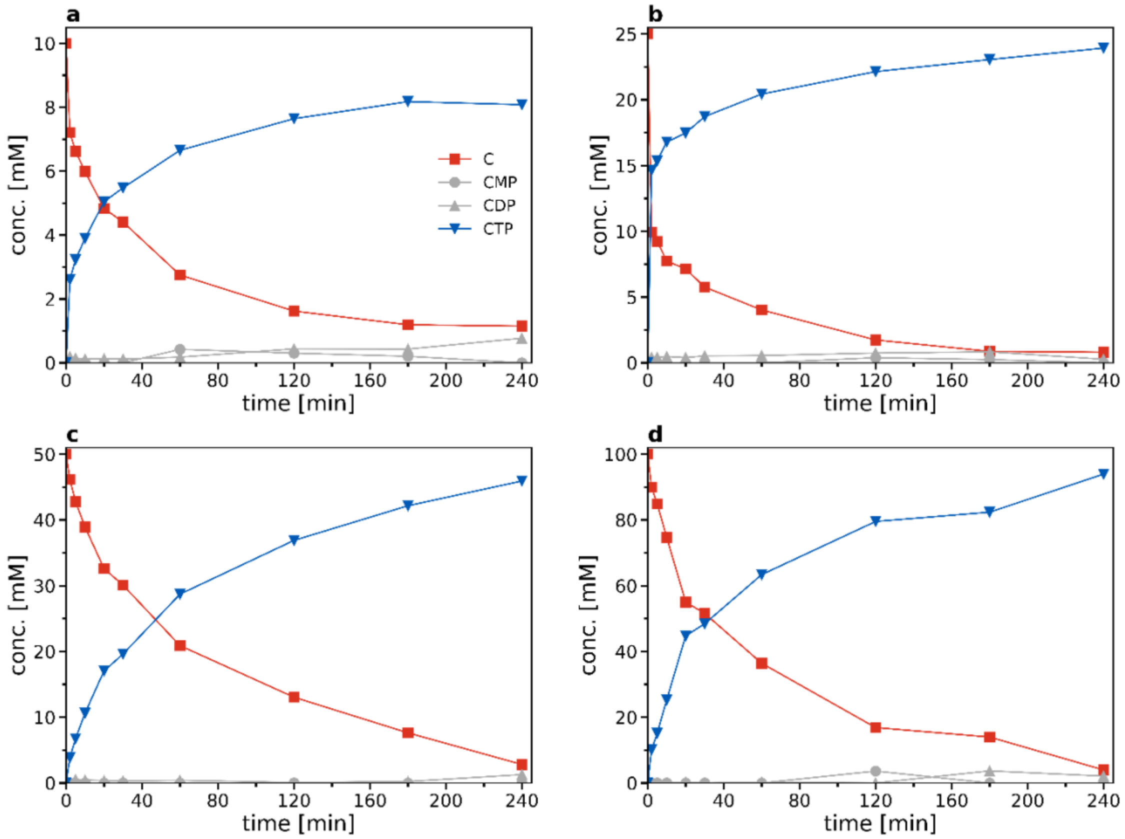

Figure S9. C cascade intensification from 10 to 100 mM (final iteration). Five mol% GTP and four equivalents of AcP were used. Enzyme loadings: **a** 10 mM C, 0.3 U mL⁻¹ *Ec*UK, 3.3 U mL⁻¹ *Sc*URA6, 13 U mL⁻¹ *Ec*AcK. **b** 25 mM C, 0.8 U mL⁻¹ *Ec*UK, 8.3 U mL⁻¹ *Sc*URA6, 33 U mL⁻¹ *Ec*AcK. **c** 50 mM C, 1.7 U mL⁻¹ *Ec*UK, 17 U mL⁻¹ *Sc*URA6, 100 U mL⁻¹ *Ec*AcK. **d** 100 mM C, 10 U mL⁻¹ *Ec*UK, 33 U mL⁻¹ *Sc*URA6, 200 U mL⁻¹ *Ec*AcK. Conditions: 1.0 mL, 50 mM TAPS (pH 8.0), 30 °C, 300 rpm.

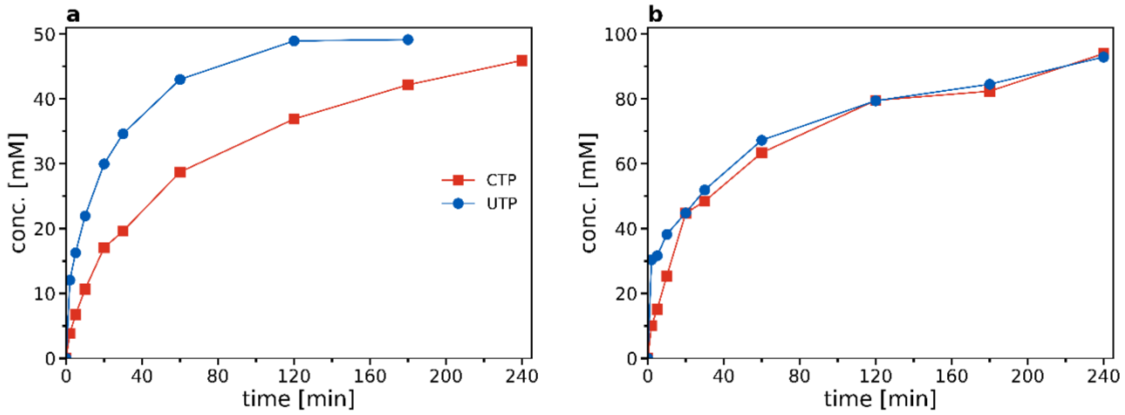

Figure S10. Comparison of C (squares) and U (circles) cascades at **a** 50 mM and **b** 100 mM substrate. Enzyme loadings (50 mM): 1.7 U mL^{-1} *Ec*UK, 17 U mL^{-1} *Sc*URA6, 100 U mL^{-1} *Ec*AcK. Enzyme loadings (100 mM): C, 10 U mL^{-1} *Ec*UK; U, 3.3 U mL^{-1} *Ec*UK; both with 33 U mL^{-1} *Sc*URA6 and 200 U mL^{-1} *Ec*AcK. Conditions: 1.0 mL, 50 mM TAPS (pH 8.0), 30 °C, 300 rpm; 50 mM $MgCl_2$; 5 mol% GTP (C) or ATP (U) as phosphate shuttle; four equivalents AcP.

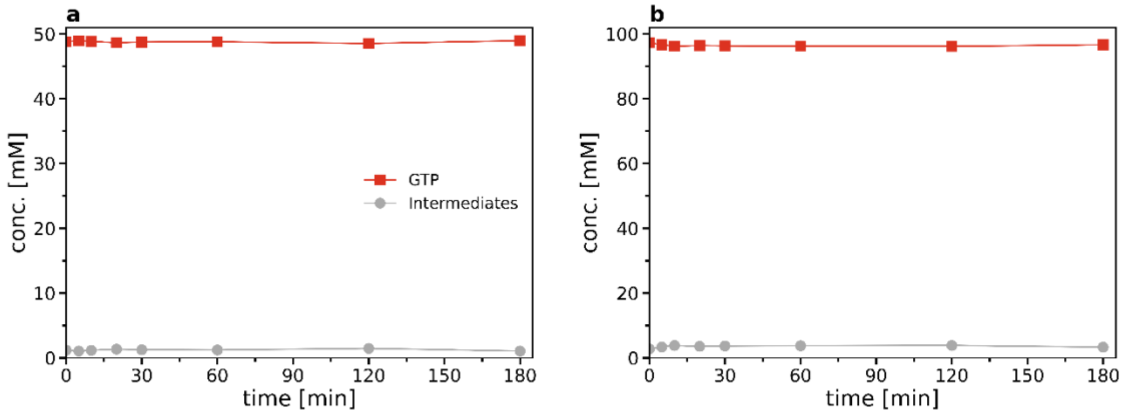

Figure S11. Conversion of G to GTP measured by RP-HPLC. Substrate loadings: **a** 50 mM and **b** 100 mM. Enzyme loadings: **a** 5 U mL^{-1} *Ea*GK, 17 U mL^{-1} *Sc*GMPK, 100 U mL^{-1} *Ec*AcK; **b** 10 U mL^{-1} *Ea*GK, 33 U mL^{-1} *Sc*GMPK, 200 U mL^{-1} *Ec*AcK. Conditions: 1.0 mL, 50 mM TAPS (pH 8.0), 30 °C, 1000 rpm; 5 mol% ATP; four equivalents AcP; 50 mM $MgCl_2$.

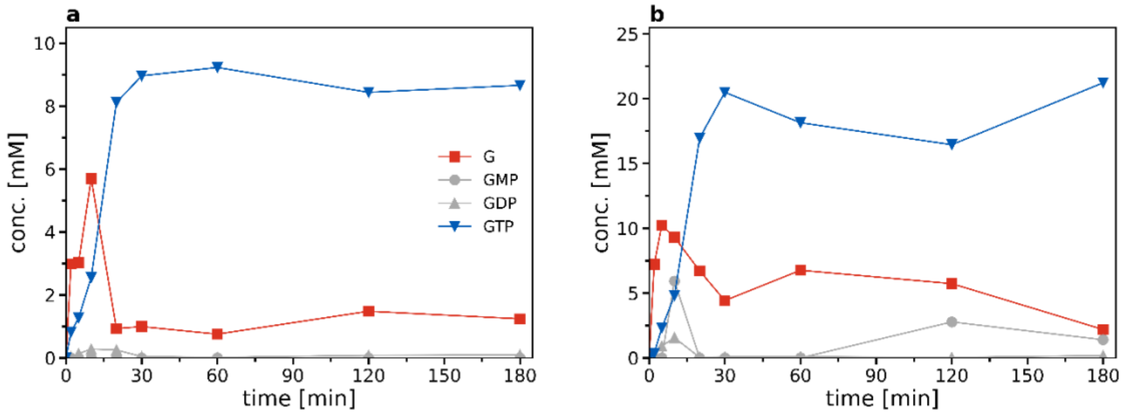

Figure S12. G cascade intensification at **a** 10 mM and **b** 25 mM. Enzyme loadings: **a** 0.3 U mL^{-1} *Ea*GK, 3.3 U mL^{-1} *Sc*GMPK, 20 U mL^{-1} *Ec*AcK; **b** 0.8 U mL^{-1} *Ea*GK, 8.3 U mL^{-1} *Sc*GMPK, 50 U mL^{-1} *Ec*AcK. Conditions: 1.0 mL, 50 mM TAPS (pH 8.0), 30 °C, 1000 rpm; 5 mol% ATP; four equivalents AcP; 50 mM $MgCl_2$.

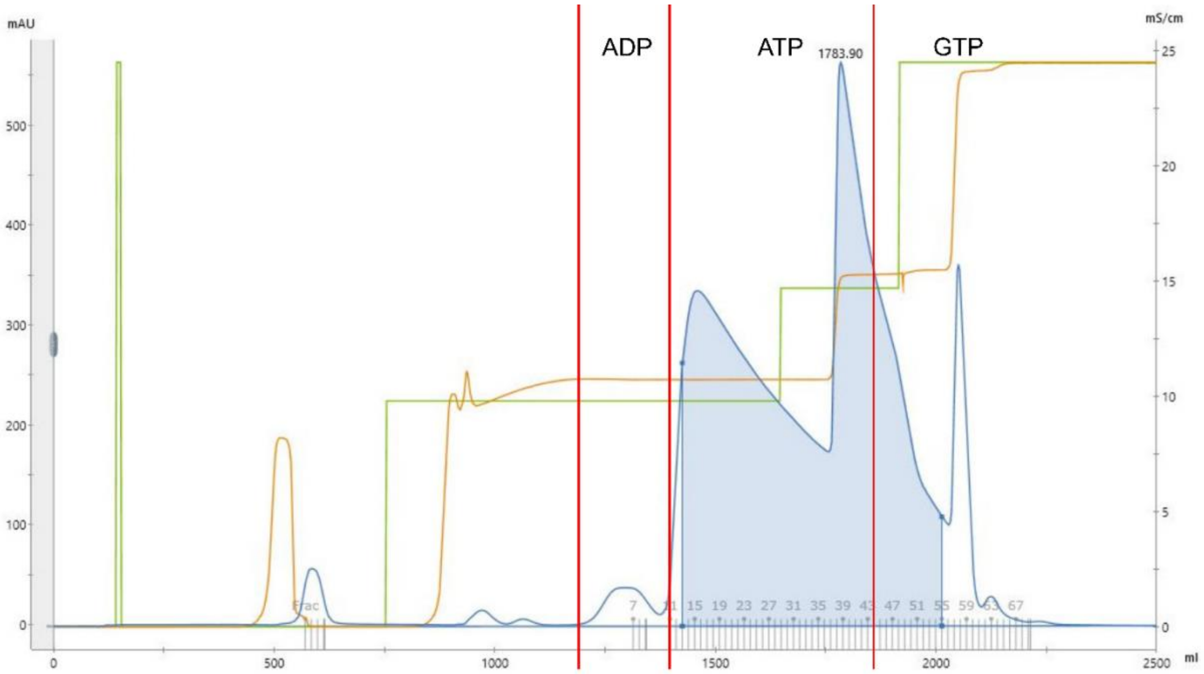

Figure S13. AEX chromatogram for ATP purification. Fractions containing ADP, ATP, and GTP are indicated. Fractions 14 – 44 were pooled for concentration. See the section 2.3.5 Preparative synthesis of NTPs in the main text for the chromatography conditions used.

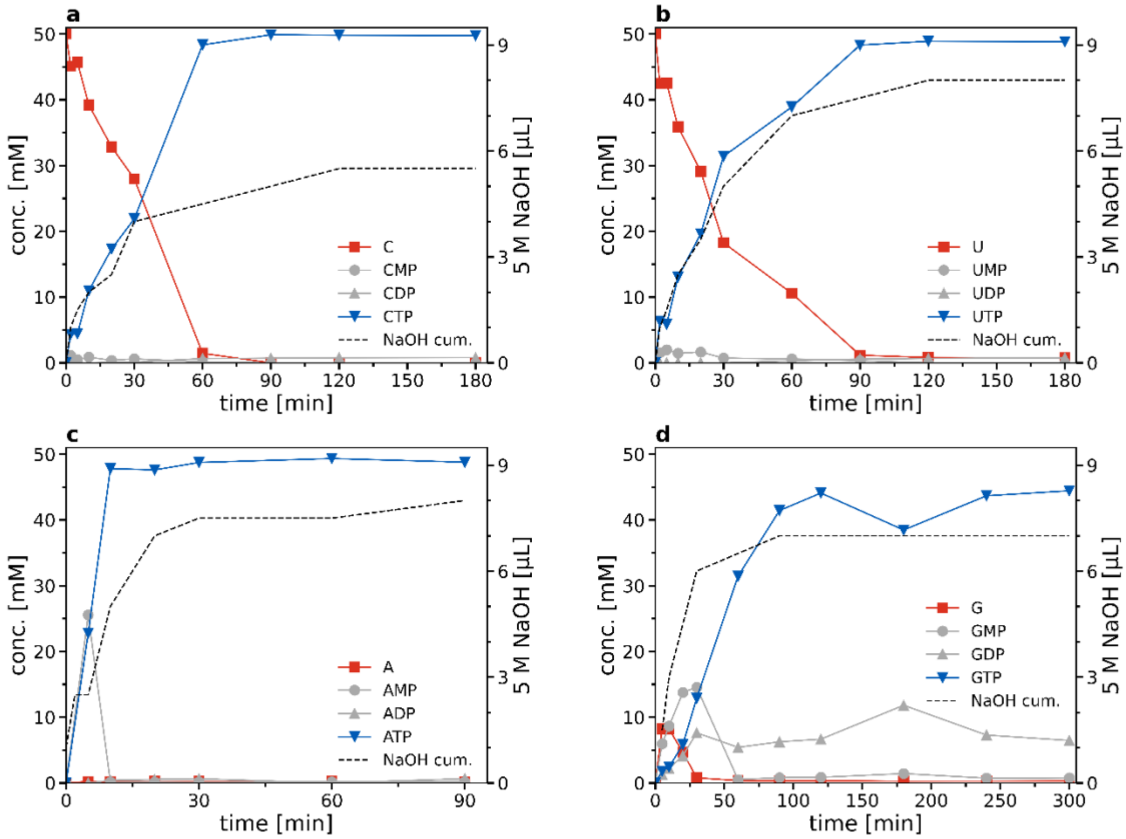

Figure S14. Auto-regeneration cascades for NTP production. Concentrations of nucleosides and nucleotides [mM] (left y-axis) and cumulative 5 M NaOH addition [µL] (right y-axis). Enzyme loadings: **a** C cascade: 5 U mL^{-1} *Ec*UK, 33 U mL^{-1} *Sc*URA6, 200 U mL^{-1} *Ec*AcK; 5.5 µL 5 M NaOH. **b** U cascade: 2.5 U mL^{-1} *Ec*UK, 17 U mL^{-1} *Sc*URA6, 100 U mL^{-1} *Ec*AcK; 8 µL 5 M NaOH. **c** A cascade: 1.7 U mL^{-1} *Sc*AK, 17 U mL^{-1} *Sc*URA6, 100 U mL^{-1} *Ec*AcK; 8 µL 5 M NaOH. **d** G cascade: 15 U mL^{-1} *Ea*GK, 50 U mL^{-1} *Sc*GMPK, 300 U mL^{-1} *Ec*AcK; 7 µL 5 M NaOH. General conditions: 1.0 mL, 50 mM TAPS (pH 8.0), 30 °C; 50 mM nucleoside; 2.5 mM target NTP (phosphate shuttle); four equivalents AcP; 50 mM $MgCl_2$; agitation at 300 rpm (C, U) or 1000 rpm (A, G).

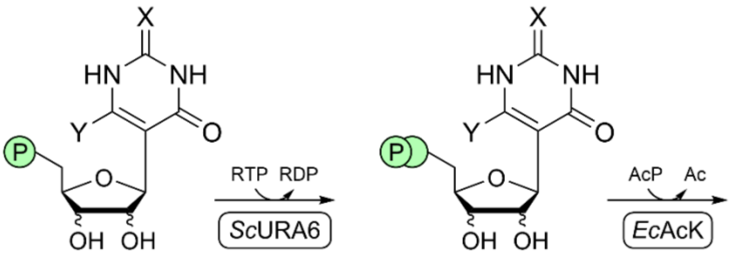

	X	Y
ΨMP	O	H
2t-ΨMP	S	H
6-amino-ΨMP	O	NH_2
araΨMP	O	H
xylΨMP	O	H

	Y_R [%]
ΨTP	81.8
2t-ΨTP	79
6-amino-ΨTP	90
araΨTP	88.8
xylΨTP	67

Figure S15. Scheme of the enzymatic phosphorylation cascade used to synthesize ΨTP and its analogues. Y_R, reaction yield in %.

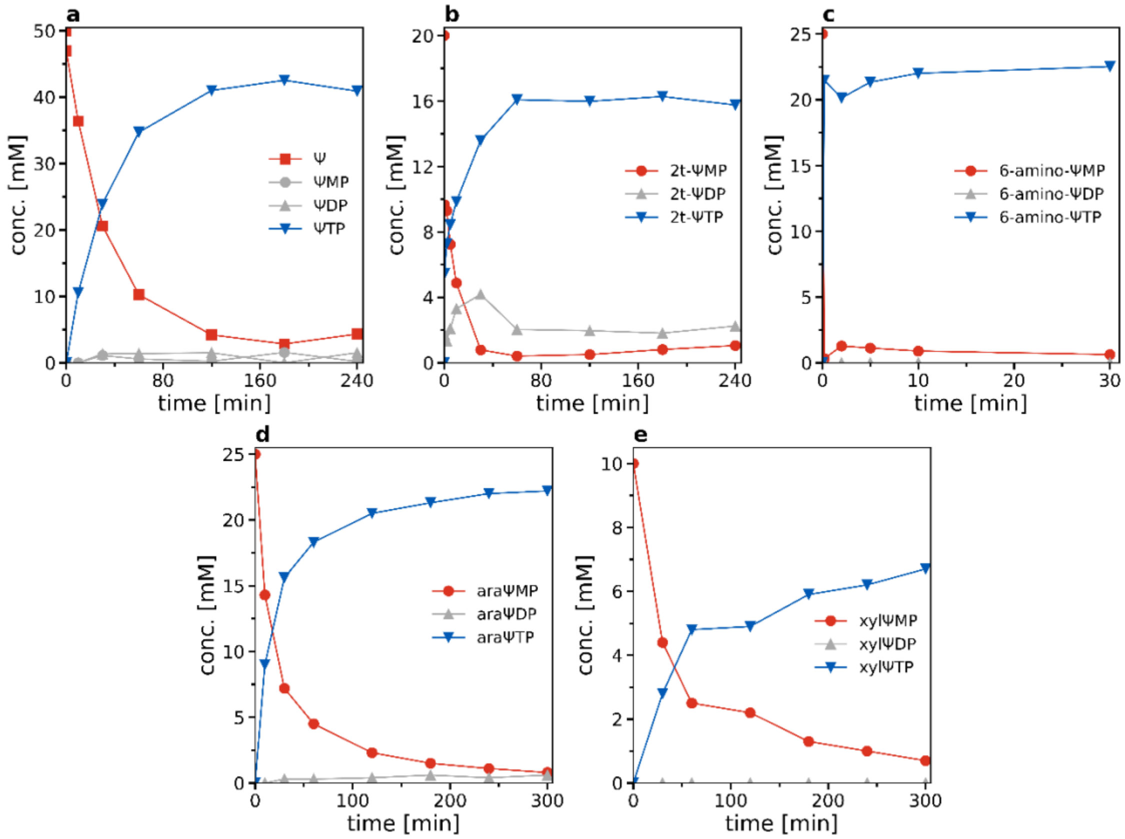

Figure S16. Time course of the enzymatic phosphorylation of ΨMP to and its analogues. **a** 2-thio-ΨTP (2t-ΨTP), **b** 6-amino-ΨTP, **c** 5-β-D-arabinofuranosyl- ΨTP (araΨTP) and **d** 5-β-D-xylofuranosyl- ΨTP (xylΨTP). The reactions were performed using substrate at 20 mM (**a**), 25 mM (**b**, **c**) and 10 mM (**d**), 5 mol% ATP and three equivalents of AcP at 30 °C and 650 rpm in 50 mM TAPS supplemented with 10 mM $MgCl_2$. **a**, **b**, **c** 0.15 mg mL^{-1} *Sc*URA6 and 0.15 mg mL^{-1} *Ec*AcK. **d** 0.3 mg mL^{-1} *Sc*URA6 and 0.3 mg mL^{-1} *Ec*AcK.

Table S1. Typical retention time (t_R) of the analyzed nucleosides and nucleotides.

Compound	t_R [min]
C / CMP / CDP / CTP	0.38 / 0.55 / 1.04 / 3.03
U / UMP / UDP / UTP	0.38 / 0.59 / 1.18 / 3.83
A / AMP / ADP / ATP	0.44 / 0.61 / 1.30 / 4.54
G / GMP / GDP / GTP	0.38 / 0.56 / 1.11 / 3.52

Table S2. Primers used in this study.

Primer name	Sequence (5' – 3')
pET-28a(+)_fwd	CACCACCACCACCACCAC
pET-28a(+)_rev	CATGGTATATCTCCTTCTTAAAGTTAAACAAAATTATTTCTAG

Table S3. Summary of cascade intensification at 1 mL scale. The table shows reaction time [min], conversion [%], and productivity [mM min^{-1}] calculated from the initial linear phase. Reactions are labeled by substrate and concentration [mM]. The symbol – indicates that the reaction was not performed.

Substrate	Substrate [mM]	Time [min]	Prod. [mM min^{-1}]	Conv. [%]
Cytidine	10 / 25 / 50 / 100	180 / 240 / 240 / 240	0.5 / 3.7 / 0.9 / 2.4	82 / 96 / 92 / 94
Uridine	- / - / 50 100	- / - / 180 / 240	- / - / 2.5 / 2.1	- / - / 98 / 93
Adenosine	10 / 25 / 50 / 100	60 / 30 / 30 / 60	0.2 / 0.6 / 0.6 / 3.5	89 / 99 / 98 / 94
Guanosine	10 / 25 / 50 / 100	180 / 180 / 60 / 60	0.4 / 0.8 / 2.0 / 3.4	87 / 85 / 98 / 97

Table S4. Summary of key parameters for NTP production from nucleosides.

	Y_R [%]	Co-substrate use [mol_{cos} mol_{sub}^{-1}]	Catalyst use [g_E g_{sub}^{-1}]	*STY* [g L^{-1} h^{-1}]
This study				
A	94	1.35	0.01	24
C	96	1.35	0.02	23
G	94	1.35	0.08	25
U	95	1.35	0.01	23
Fehlau, 2020[2]				
A	99	2.9	0.8	0.03
C	97	2.9	0.9	0.02
Benčić, 2023[3]				
A	66	3.3	0.5	0.06
C	8	3.3	0.5	0.006
G	62	3.3	0.5	0.05
U	54	3.3	0.5	0.04
Meng, 2025[4]				
2'-MOE-A	76	7.5	0.8	0.08
2'-F-A	77	9.2	1.1	0.05

Comparative reaction conditions: This study: 10 mL, 1 h (2 h for C), 50 mM substrate, 50 mM $MgCl_2$, 30 °C, pH 8.0. Fehlau et al.: 5 µL, 19 h, 1 mM substrate, 5 mM $MgCl_2$, 37 °C, pH 7.6. Benčić et al.: 1 mL, 24 h, 4 mM substrate, 20 mM $MgCl_2$, 37 °C, pH 8.0. Meng et al.: 65 mL/450 mL (2'-MOE-A and 2'-F-A respectively), 44 h, 55 mM $MgCl_2$, 30 °C, pH 4 (first step), pH 7 (subsequent steps). Abbreviations: Y_R [%], reaction yield; cosub. [mol_{cos} mol_{sub}^{-1}], molar ratio of co-substrate to substrate per reaction step; catalyst [g_E g_{sub}^{-1}], total catalyst mass per substrate mass; *STY* [g L^{-1} h^{-1}], space time yield calculated over the total reaction time.

Table S5. Reported additional substrates for enzymes used in this study (excluding the substrates applied here).

Enzymes	Reported additional substrates
*Ea*GK	3'-O-methyl-G [5], 3'-amino-ddG [6†]
*Ec*UK	3-F-C [7], 5-azaC [8], 5-methyl-U [9†], Ψ [10†], m1Ψ [11]
*Sc*AK	2-F-A, ara-A, dA [2†]
*Sc*GMPK	2-F-AMP, dAMP [2†]; 3'-O-methyl-GMP [5†], 8-azaGMP [12†]
*Sc*URA6	5-I-dUMP [13]; dCMP, ΨMP, 2t-ΨMP, 6-amino-ΨMP, araΨMP, xylΨMP [14]
*Ec*AcK	dCDP, 2t-ΨDP, 6-amino-ΨDP, araΨDP, xylΨDP [14]; ΨDP [10,14‡], m1ΨDP [11,14‡], 3'-NH_2ddGDP [6†]

† Activity reported for a homolog from a different organism. ‡ Activity reported for the variant used in this study and for homologous enzymes. Abbreviations: 2tΨMP/DP, 2 thio pseudouridine 5′ monophosphate/diphosphate; 5 azaC, 5 azacytidine; 8 azaGMP, 8 azaguanosine 5′ monophosphate; ara A, vidarabine; ara ΨMP/DP, 5 β D arabinofuranosyl Ψ-5′-monophosphate/diphosphate; ddG, 2′,3′ dideoxyguanosine; m1Ψ, N1 methyl-Ψ; xyl ΨMP/DP, 5 β D xylofuranosyl-Ψ-5′ monophosphate/diphosphate; Ψ, pseudouridine.

Gene and plasmid sequences

3.5. pET-15b(+)

3.5.1. vector sequence

```
>pET-15b(+) empty vector
```

```
TTCTCATGTTTGACAGCTTATCATCGATAAGCTTTAATGCGGTAGTTTATCACAGTTAAATTGCTAACGCAGTCA
GGCACCGTGTATGAAATCTAACAATGCGCTCATCGTCATCCTCGGCACCGTCACCCTGGATGCTGTAGGCATAGG
CTTGGTTATGCCGGTACTGCCGGGCCTCTTGCGGGATATCCGGATATAGTTCCTCCTTTCAGCAAAAAACCCCTC
AAGACCCGTTTAGAGGCCCCAAGGGGTTATGCTAGTTATTGCTCAGCGGTGGCAGCAGCCAACTCAGCTTCCTTT
CGGGCTTTGTTAGCAGCCGGATCCTCGAGCATATGGCTGCCGCGCGGCACCAGGCCGCTGCTGTGATGATGATGA
TGATGGCTGCTGCCCATGGTATATCTCCTTCTTAAAGTTAAACAAAATTATTTCTAGAGGGGAATTGTTATCCGC
TCACAATTCCCCTATAGTGAGTCGTATTAATTTCGCGGGATCGAGATCTCGATCCTCTACGCCGGACGCATCGTG
GCCGGCATCACCGGCGCCACAGGTGCGGTTGCTGGCGCCTATATCGCCGACATCACCGATGGGGAAGATCGGGCT
CGCCACTTCGGGCTCATGAGCGCTTGTTTCGGCGTGGGTATGGTGGCAGGCCCCGTGGCCGGGGGACTGTTGGGC
GCCATCTCCTTGCATGCACCATTCCTTGCGGCGGCGGTGCTCAACGGCCTCAACCTACTACTGGGCTGCTTCCTA
ATGCAGGAGTCGCATAAGGGAGAGCGTCGAGATCCCGGACACCATCGAATGGCGCAAAACCTTTCGCGGTATGGC
ATGATAGCGCCCGGAAGAGAGTCAATTCAGGGTGGTGAATGTGAAACCAGTAACGTTATACGATGTCGCAGAGTA
TGCCGGTGTCTCTTATCAGACCGTTTCCCGCGTGGTGAACCAGGCCAGCCACGTTTCTGCGAAAACGCGGGAAAA
AGTGGAAGCGGCGATGGCGGAGCTGAATTACATTCCCAACCGCGTGGCACAACAACTGGCGGGCAAACAGTCGTT
GCTGATTGGCGTTGCCACCTCCAGTCTGGCCCTGCACGCGCCGTCGCAAATTGTCGCGGCGATTAAATCTCGCGC
CGATCAACTGGGTGCCAGCGTGGTGGTGTCGATGGTAGAACGAAGCGGCGTCGAAGCCTGTAAAGCGGCGGTGCA
CAATCTTCTCGCGCAACGCGTCAGTGGGCTGATCATTAACTATCCGCTGGATGACCAGGATGCCATTGCTGTGGA
AGCTGCCTGCACTAATGTTCCGGCGTTATTTCTTGATGTCTCTGACCAGACACCCATCAACAGTATTATTTTCTC
CCATGAAGACGGTACGCGACTGGGCGTGGAGCATCTGGTCGCATTGGGTCACCAGCAAATCGCGCTGTTAGCGGG
CCCATTAAGTTCTGTCTCGGCGCGTCTGCGTCTGGCTGGCTGGCATAAATATCTCACTCGCAATCAAATTCAGCC
GATAGCGGAACGGGAAGGCGACTGGAGTGCCATGTCCGGTTTTCAACAAACCATGCAAATGCTGAATGAGGGCAT
CGTTCCCACTGCGATGCTGGTTGCCAACGATCAGATGGCGCTGGGCGCAATGCGCGCCATTACCGAGTCCGGGCT
GCGCGTTGGTGCGGATATCTCGGTAGTGGGATACGACGATACCGAAGACAGCTCATGTTATATCCCGCCGTTAAC
CACCATCAAACAGGATTTTCGCCTGCTGGGGCAAACCAGCGTGGACCGCTTGCTGCAACTCTCTCAGGGCCAGGC
GGTGAAGGGCAATCAGCTGTTGCCCGTCTCACTGGTGAAAAGAAAAACCACCCTGGCGCCCAATACGCAAACCGC
CTCTCCCCGCGCGTTGGCCGATTCATTAATGCAGCTGGCACGACAGGTTTCCCGACTGGAAAGCGGGCAGTGAGC
GCAACGCAATTAATGTAAGTTAGCTCACTCATTAGGCACCGGGATCTCGACCGATGCCCTTGAGAGCCTTCAACC
CAGTCAGCTCCTTCCGGTGGGCGCGGGGCATGACTATCGTCGCCGCACTTATGACTGTCTTCTTTATCATGCAAC
TCGTAGGACAGGTGCCGGCAGCGCTCTGGGTCATTTTCGGCGAGGACCGCTTTCGCTGGAGCGCGACGATGATCG
GCCTGTCGCTTGCGGTATTCGGAATCTTGCACGCCCTCGCTCAAGCCTTCGTCACTGGTCCCGCCACCAAACGTT
TCGGCGAGAAGCAGGCCATTATCGCCGGCATGGCGGCCGACGCGCTGGGCTACGTCTTGCTGGCGTTCGCGACGC
GAGGCTGGATGGCCTTCCCCATTATGATTCTTCTCGCTTCCGGCGGCATCGGGATGCCCGCGTTGCAGGCCATGC
TGTCCAGGCAGGTAGATGACGACCATCAGGGACAGCTTCAAGGATCGCTCGCGGCTCTTACCAGCCTAACTTCGA
TCACTGGACCGCTGATCGTCACGGCGATTTATGCCGCCTCGGCGAGCACATGGAACGGGTTGGCATGGATTGTAG
GCGCCGCCCTATACCTTGTCTGCCTCCCCGCGTTGCGTCGCGGTGCATGGAGCCGGGCCACCTCGACCTGAATGG
AAGCCGGCGGCACCTCGCTAACGGATTCACCACTCCAAGAATTGGAGCCAATCAATTCTTGCGGAGAACTGTGAA
TGCGCAAACCAACCCTTGGCAGAACATATCCATCGCGTCCGCCATCTCCAGCAGCCGCACGCGGCGCATCTCGGG
CAGCGTTGGGTCCTGGCCACGGGTGCGCATGATCGTGCTCCTGTCGTTGAGGACCCGGCTAGGCTGGCGGGGTTG
CCTTACTGGTTAGCAGAATGAATCACCGATACGCGAGCGAACGTGAAGCGACTGCTGCTGCAAAACGTCTGCGAC
CTGAGCAACAACATGAATGGTCTTCGGTTTCCGTGTTTCGTAAAGTCTGGAAACGCGGAAGTCAGCGCCCTGCAC
CATTATGTTCCGGATCTGCATCGCAGGATGCTGCTGGCTACCCTGTGGAACACCTACATCTGTATTAACGAAGCG
CTGGCATTGACCCTGAGTGATTTTTCTCTGGTCCCGCCGCATCCATACCGCCAGTTGTTTACCCTCACAACGTTC
CAGTAACCGGGCATGTTCATCATCAGTAACCCGTATCGTGAGCATCCTCTCTCGTTTCATCGGTATCATTACCCC
CATGAACAGAAATCCCCCTTACACGGAGGCATCAGTGACCAAACAGGAAAAAACCGCCCTTAACATGGCCCGCTT
TATCAGAAGCCAGACATTAACGCTTCTGGAGAAACTCAACGAGCTGGACGCGGATGAACAGGCAGACATCTGTGA
ATCGCTTCACGACCACGCTGATGAGCTTTACCGCAGCTGCCTCGCGCGTTTCGGTGATGACGGTGAAAACCTCTG
ACACATGCAGCTCCCGGAGACGGTCACAGCTTGTCTGTAAGCGGATGCCGGGAGCAGACAAGCCCGTCAGGGCGC
GTCAGCGGGTGTTGGCGGGTGTCGGGGCGCAGCCATGACCCAGTCACGTAGCGATAGCGGAGTGTATACTGGCTT
AACTATGCGGCATCAGAGCAGATTGTACTGAGAGTGCACCATATATGCGGTGTGAAATACCGCACAGATGCGTAA
GGAGAAAATACCGCATCAGGCGCTCTTCCGCTTCCTCGCTCACTGACTCGCTGCGCTCGGTCGTTCGGCTGCGGC
GAGCGGTATCAGCTCACTCAAAGGCGGTAATACGGTTATCCACAGAATCAGGGGATAACGCAGGAAAGAACATGT
```

GAGCAAAAGGCCAGCAAAAGGCCAGGAACCGTAAAAAGGCCGCGTTGCTGGCGTTTTTCCATAGGCTCCGCCCCC
CTGACGAGCATCACAAAAATCGACGCTCAAGTCAGAGGTGGCGAAACCCGACAGGACTATAAAGATACCAGGCGT
TTCCCCCTGGAAGCTCCCTCGTGCGCTCTCCTGTTCCGACCCTGCCGCTTACCGGATACCTGTCCGCCTTTCTCC
CTTCGGGAAGCGTGGCGCTTTCTCATAGCTCACGCTGTAGGTATCTCAGTTCGGTGTAGGTCGTTCGCTCCAAGC
TGGGCTGTGTGCACGAACCCCCCGTTCAGCCCGACCGCTGCGCCTTATCCGGTAACTATCGTCTTGAGTCCAACC
CGGTAAGACACGACTTATCGCCACTGGCAGCAGCCACTGGTAACAGGATTAGCAGAGCGAGGTATGTAGGCGGTG
CTACAGAGTTCTTGAAGTGGTGGCCTAACTACGGCTACACTAGAAGGACAGTATTTGGTATCTGCGCTCTGCTGA
AGCCAGTTACCTTCGGAAAAAGAGTTGGTAGCTCTTGATCCGGCAAACAAACCACCGCTGGTAGCGGTGGTTTTT
TTGTTTGCAAGCAGCAGATTACGCGCAGAAAAAAAGGATCTCAAGAAGATCCTTTGATCTTTTCTACGGGGTCTG
ACGCTCAGTGGAACGAAAACTCACGTTAAGGGATTTTGGTCATGAGATTATCAAAAAGGATCTTCACCTAGATCC
TTTTAAATTAAAAATGAAGTTTTAAATCAATCTAAAGTATATATGAGTAAACTTGGTCTGACAGTTACCAATGCT
TAATCAGTGAGGCACCTATCTCAGCGATCTGTCTATTTCGTTCATCCATAGTTGCCTGACTCCCCGTCGTGTAGA
TAACTACGATACGGGAGGGCTTACCATCTGGCCCCAGTGCTGCAATGATACCGCGAGACCCACGCTCACCGGCTC
CAGATTTATCAGCAATAAACCAGCCAGCCGGAAGGGCCGAGCGCAGAAGTGGTCCTGCAACTTTATCCGCCTCCA
TCCAGTCTATTAATTGTTGCCGGGAAGCTAGAGTAAGTAGTTCGCCAGTTAATAGTTTGCGCAACGTTGTTGCCA
TTGCTGCAGGCATCGTGGTGTCACGCTCGTCGTTTGGTATGGCTTCATTCAGCTCCGGTTCCCAACGATCAAGGC
GAGTTACATGATCCCCCATGTTGTGCAAAAAAGCGGTTAGCTCCTTCGGTCCTCCGATCGTTGTCAGAAGTAAGT
TGGCCGCAGTGTTATCACTCATGGTTATGGCAGCACTGCATAATTCTCTTACTGTCATGCCATCCGTAAGATGCT
TTTCTGTGACTGGTGAGTACTCAACCAAGTCATTCTGAGAATAGTGTATGCGGCGACCGAGTTGCTCTTGCCCGG
CGTCAACACGGGATAATACCGCGCCACATAGCAGAACTTTAAAAGTGCTCATCATTGGAAAACGTTCTTCGGGGC
GAAAACTCTCAAGGATCTTACCGCTGTTGAGATCCAGTTCGATGTAACCCACTCGTGCACCCAACTGATCTTCAG
CATCTTTTACTTTCACCAGCGTTTCTGGGTGAGCAAAAACAGGAAGGCAAAATGCCGCAAAAAAGGGAATAAGGG
CGACACGGAAATGTTGAATACTCATACTCTTCCTTTTTCAATATTATTGAAGCATTTATCAGGGTTATTGTCTCA
TGAGCGGATACATATTTGAATGTATTTAGAAAAATAAACAAATAGGGGTTCCGCGCACATTTCCCCGAAAAGTGC
CACCTGACGTCTAAGAAACCATTATTATCATGACATTAACCTATAAAAATAGGCGTATCACGAGGCCCTTTCGTC
TTCAAGAA

3.5.2. *Ec*UK

>X71492.1 E.coli udk gene for uridine/cytidine kinase

GAATTCTTGTCAACAATTAACTCTGAGCGCGCGGCAACGCTATTCGACTGGTATCAGACGGATGAAATCCCTATA
ATTGCCGCGTTTGGCGCTTCGTCGCCCCCTTCCTAACATCCAGGTTAATCAGGTCGCTAAATATATGACTGATCA
GTCTCATCAGTGCGTCATTATCGGTATCGCTGGCGCATCGGCTTCCGGCAAGAGTCTTATTGCCAGTACCCTTTA
TCGTGAATTGCGTGAGCAAGTCGGTGATGAACACATCGGCGTAATTCCCGAAGACTGCTATTACAAAGATCAAAG
CCATCTGTCGATGGAAGAACGCGTTAAGACCAACTACGACCATCCCAGCGCGATGGATCACAGTCTGCTGCTTGA
GCATTTACAAGCGTTGAAACGCGGCTCGGCAATTGACCTGCCGGTTTACAGCTATGTTGAACATACGCGTATGAA
AGAAACGGTGACGGTTGAGCCGAAGAAGGTCATCATTCTCGAAGGCATTTTGTTGCTGACGGATGCGCGTTTGCG
TGACGAACTTAACTTCTCCATTTTCGTTGATACCCCGCTGGATATCTGCCTGATGCGCCGCATCAAGCGTGACGT
TAACGAGCGTGGGCGTTCAATGGATTCAGTGATGGCGCAATATCAAAAAACCGTGCGCCCGATGTTCCTGCAATT
CATTGAGCCTTCTAAACAATATGCGGACATTATCGTGCCGCGCGGCGGGAAAAACCGCATCGCGATCGATATATT
GAAAGCGAAAATAAGTCAGTTCTTTGAATAAGCTT

>sp|P0A8F4|URK_ECOLI Uridine kinase OS=Escherichia coli (strain K12) OX=83333 GN=udk PE=3 SV=1 + N terminal His_6-tag

HHHHHHMTDQSHQCVIIGIAGASASGKSLIASTLYRELREQVGDEHIGVIPEDCYYKDQSHLSMEERVKTNYDHP
SAMDHSLLLEHLQALKRGSAIDLPVYSYVEHTRMKETVTVEPKKVIILEGILLLTDARLRDELNFSIFVDTPLDI
CLMRRIKRDVNERGRSMDSVMAQYQKTVRPMFLQFIEPSKQYADIIVPRGGKNRIAIDILKAKISQFFE

3.5.3. *Sc*URA6

>M69295.1 Saccharomyces cerevisiae uridine monophosphate kinase (URA6) gene, complete cds

ACGCTACAGGTTTATACTGAGATTTTATTCTTCGAAAAAACCAACTTTCCACAATACAGCACCTTCGAAGACCAA
CGTAAACGTACCACGCGCTAACAAAAGTCAAAGCAAGGGTAAACACAAAGGCAAATTGTTGGTACTCGTAGGAAC
TTTAGCACTCGTGACATCAGTTATTTCGGTAAACTATCAAAAGAACGAACCAGTTGAATTTCTTGAATAAGACAA
ATGACAGCTGCCACTACATCACAGCCAGCTTTCTCGCCTGACCAAGTTTCCGTGATCTTCGTTCTAGGAGGACCC
GGTGCAGGCAAGGGTACTCAGTGTGAAAAACTAGTTAAGGACTATTCATTTGTCCATTTGTCAGCCGGAGACCTT
CTACGTGCTGAGCAGGGCAGAGCAGGTTCCCAATATGGGGAATTGATCAAGAACTGCATCAAAGAGGGCCAGATT
GTCCCTCAAGAGATTACTTTGGCGCTTTTACGCAACGCTATTTCCGATAACGTCAAGGCGAACAAGCATAAGTTC
TTAATTGACGGATTTCCTAGGAAGATGGATCAAGCCATTTCCTTTGAAAGAGACATCGTTGAAAGCAAATTCATC
CTGTTCTTTGACTGCCCTGAAGATATCATGTTAGAGAGACTATTGGAGCGTGGCAAGACCAGTGGTAGAAGCGAT

GACAACATTGAGTCCATTAAGAAGAGATTTAACACTTTCAAGGAGACTAGTATGCCCGTCATCGAGTACTTTGAA
ACCAAATCGAAAGTCGTCCGTGTTCGTTGCGACAGATCCGTCGAAGATGTGTACAAAGACGTCCAAGACGCTATC
CGTGATAGCTTATAGACTAAGACGTATTTTATATAGTTTACGCTATAAGTTACGGCGATATTATATATATATGTA
AGAATATATACATATATATTAAAATGATTATATGCCCTCCCCTACATATCAGACCTTTACAGGGTAACCTTAAAA
TAAACTGCTAAATTATTTGGTTTATGAATTAAAAGAGCAATAATAACAATGGCGGACGACTGAAACGATGATAG
GTAAGCCTAAAAGACTAGTCAGACGTATCTTACGCCATGGACAAAATCAATCCTGATTGGGCGAAGGATATCCGT
GCAGAAATATCACTATTTATGGCTACTCGAAAAAGGAGAAAGAAGGTTGCC

>sp|P15700|KCY_YEAST Uridylate kinase OS=Saccharomyces cerevisiae (strain ATCC 204508 / S288c) OX=559292 GN=URA6 PE=1 SV=1 + N terminal His$_6$-tag

HHHHHHMTAATTSQPAFSPDQVSVIFVLGGPGAGKGTQCEKLVKDYSFVHLSAGDLLRAEQGRAGSQYGELIKNC
IKEGQIVPQEITLALLRNAISDNVKANKHKFLIDGFPRKMDQAISFERDIVESKFILFFDCPEDIMLERLLERGK
TSGRSDDNIESIKKRFNTFKETSMPVIEYFETKSKVVRVRCDRSVEDVYKDVQDAIRDSL

3.5.4. *Ec*AcK

>M22956.1 E.coli ackA gene encoding acetate kinase, complete cds

ACGCGTCATCTTGATAACGCGATTTTCGACAAAGACCGGGGCAAGGCGTTTTTCCAGCGGCCACGTCTTTGAGTA
ATGCTGTCCCCGGCGAAACAAGCTAAAAAAATTAACAGAACGATTATCCGGCGTTGACATGCTTCACCTCAACTT
CACATATAAAGATTCAAAATTTGTGCAAATTCACAACTCAGCGGGACAACGTTCAAAACATTTTGTCTTCCATAC
CCACTATCAGGTATCCTTTAGCAGCCTGAAGGCCTAAGTAGTACATATTCATTGAGTCGTCAAATTCATATACAT
TATGCCATTGGCTGAAAATTACGCAAAATGGCATAGACTCAAGATATTTCTTCCATCATGCAAAAAAAATTTGCA
GTGCATGATGTTAATCATAAATGTCGGTGTCATCATGCGCTACGCTCTATGGCTCCCTGACGTTTTTTTAGCCAC
GTATCAATTATAGGTACTTCCATGTCGAGTAAGTTAGTACTGGTTCTGAACTGCGGTAGTTCTTCACTGAAATTT
GCCATCATCGATGCAGTAAATGGTGAAGAGTACCTTTCTGGTTTAGCCGAATGTTTCCACCTGCCCGAAGCACGT
ATCAAATGGAAAATGGACGGCAATAAACAGGAAGCGGCTTTAGGTGCAGGCGCCGCTCACAGCGAAGCGCTCAAC
TTTATCGTTAATACTATTCTGGCACAAAAACCAGAACTGTCTGCGCAGCTGACTGCTATCGGTCACCGTATCGTA
CACGGCGGCGAAAAGTATACCAGCTCCGTAGTGATCGATGAGTCTGTTATTCAGGGTATCAAAGATGCAGCTTCT
TTTGCACCGCTGCACAACCCGGCTCACCTGATCGGTATCGAAGAAGCTCTGAAATCTTTCCCACAGCTGAAAGAC
AAAAACGTTGCTGTATTTGACACCGCGTTCCACCAGACTATGCCGGAAGAGTCTTACCTCTACGCCCTGCCTTAC
AACCTGTACAAAGAGCACGGCATCCGTCGTTACGGCGCGCACGGCACCAGCCACTTCTATGTAACCCAGGAAGCG
GCAAAAATGCTGAACAAACCGGTAGAAGAACTGAACATCATCACCTGCCACCTGGGCAACGGTGGTTCCGTTTCT
GCTATCCGCAACGGTAAATGCGTTGACACCTCTATGGGCCTGACCCCGCTGGAAGGTCTGGTCATGGGTACCCGT
TCTGGTGATATCGATCCGGCGATCATCTTCCACCTGCACGACACCCTGGGCATGAGCGTTGACGCAATCAACAAA
CTGCTGACCAAAGAGTCTGGCCTGCTGGGTCTGACCGAAGTGACCAGCGACTGCCGCTATGTTGAAGACAACTAC
GCGACGAAAGAAGACGCGAAGCGCGCAATGGACGTTTACTGCCACCGCCTGGCGAAATACATCGGTGCCTACACT
GCGCTGATGGATGGTCGTCTGGACGCTGTTGTATTCACTGGTGGTATCGGTGAAAATGCCGCAATGGTTCGTGAA
CTGTCTCTGGGCAAACTGGGCGTGCTGGGCTTTGAAGTTGATCATGAACGCAACCTGGCTGCACGTTTCGGCAAA
TCTGGTTTCATCAACAAAGAAGGTACCCGTCCTGCGGTGGTTATCCCAACCAACGAAGAACTGGTTATCGCGCAA
GACGCGAGCCGCCTGACTGCCTGATTTCACACGCCAGCTCAGCTGTGTGTTTTGTAACCCGCCAAATCGGCGTTA
ACGAAAGAGGATAAACCGTGTCCCGTATTATTA

>sp|P0A6A3|ACKA_ECOLI Acetate kinase OS=Escherichia coli (strain K12) OX=83333 GN=ackA PE=1 SV=1 + N terminal His$_6$-tag

HHHHHHMSSKLVLVLNCGSSSLKFAIIDAVNGEEYLSGLAECFHLPEARIKWKMDGNKQEAALGAGAAHSEALNF
IVNTILAQKPELSAQLTAIGHRIVHGGEKYTSSVVIDESVIQGIKDAASFAPLHNPAHLIGIEEALKSFPQLKDK
NVAVFDTAFHQTMPEESYLYALPYNLYKEHGIRRYGAHGTSHFYVTQEAAKMLNKPVEELNIITCHLGNGGSVSA
IRNGKCVDTSMGLTPLEGLVMGTRSGDIDPAIIFHLHDTLGMSVDAINKLLTKESGLLGLTEVTSDCRYVEDNYA
TKEDAKRAMDVYCHRLAKYIGAYTALMDGRLDAVVFTGGIGENAAMVRELSLGKLGVLGFEVDHERNLAARFGKS
GFINKEGTRPAVVIPTNEELVIAQDASRLTA

3.6. pET-28a(+)

3.6.1. vector sequence

>pET-28a(+) empty vector

TGGCGAATGGGACGCGCCCTGTAGCGGCGCATTAAGCGCGGCGGGTGTGGTGGTTACGCGCAGCGTGACCGCTAC
ACTTGCCAGCGCCCTAGCGCCCGCTCCTTTCGCTTTCTTCCCTTCCTTTCTCGCCACGTTCGCCGGCTTTCCCCG

TCAAGCTCTAAATCGGGGGCTCCCTTTAGGGTTCCGATTTAGTGCTTTACGGCACCTCGACCCCAAAAAACTTGA
TTAGGGTGATGGTTCACGTAGTGGGCCATCGCCCTGATAGACGGTTTTTCGCCCTTTGACGTTGGAGTCCACGTT
CTTTAATAGTGGACTCTTGTTCCAAACTGGAACAACACTCAACCCTATCTCGGTCTATTCTTTTGATTTATAAGG
GATTTTGCCGATTTCGGCCTATTGGTTAAAAAATGAGCTGATTTAACAAAAATTTAACGCGAATTTTAACAAAAT
ATTAACGTTTACAATTTCAGGTGGCACTTTTCGGGGAAATGTGCGCGGAACCCCTATTTGTTTATTTTTCTAAAT
ACATTCAAATATGTATCCGCTCATGAATTAATTCTTAGAAAAACTCATCGAGCATCAAATGAAACTGCAATTTAT
TCATATCAGGATTATCAATACCATATTTTTGAAAAAGCCGTTTCTGTAATGAAGGAGAAAACTCACCGAGGCAGT
TCCATAGGATGGCAAGATCCTGGTATCGGTCTGCGATTCCGACTCGTCCAACATCAATACAACCTATTAATTTCC
CCTCGTCAAAAATAAGGTTATCAAGTGAGAAATCACCATGAGTGACGACTGAATCCGGTGAGAATGGCAAAAGTT
TATGCATTTCTTTCCAGACTTGTTCAACAGGCCAGCCATTACGCTCGTCATCAAAATCACTCGCATCAACCAAAC
CGTTATTCATTCGTGATTGCGCCTGAGCGAGACGAAATACGCGATCGCTGTTAAAAGGACAATTACAAACAGGAA
TCGAATGCAACCGGCGCAGGAACACTGCCAGCGCATCAACAATATTTTCACCTGAATCAGGATATTCTTCTAATA
CCTGGAATGCTGTTTTCCCGGGGATCGCAGTGGTGAGTAACCATGCATCATCAGGAGTACGGATAAAATGCTTGA
TGGTCGGAAGAGGCATAAATTCCGTCAGCCAGTTTAGTCTGACCATCTCATCTGTAACATCATTGGCAACGCTAC
CTTTGCCATGTTTCAGAAACAACTCTGGCGCATCGGGCTTCCCATACAATCGATAGATTGTCGCACCTGATTGCC
CGACATTATCGCGAGCCCATTTATACCCATATAAATCAGCATCCATGTTGGAATTTAATCGCGGCCTAGAGCAAG
ACGTTTCCCGTTGAATATGGCTCATAACACCCCTTGTATTACTGTTTATGTAAGCAGACAGTTTTATTGTTCATG
ACCAAAATCCCTTAACGTGAGTTTTCGTTCCACTGAGCGTCAGACCCCGTAGAAAAGATCAAAGGATCTTCTTGA
GATCCTTTTTTTCTGCGCGTAATCTGCTGCTTGCAAACAAAAAAACCACCGCTACCAGCGGTGGTTTGTTTGCCG
GATCAAGAGCTACCAACTCTTTTTCCGAAGGTAACTGGCTTCAGCAGAGCGCAGATACCAAATACTGTCCTTCTA
GTGTAGCCGTAGTTAGGCCACCACTTCAAGAACTCTGTAGCACCGCCTACATACCTCGCTCTGCTAATCCTGTTA
CCAGTGGCTGCTGCCAGTGGCGATAAGTCGTGTCTTACCGGGTTGGACTCAAGACGATAGTTACCGGATAAGGCG
CAGCGGTCGGGCTGAACGGGGGGTTCGTGCACACAGCCCAGCTTGGAGCGAACGACCTACACCGAACTGAGATAC
CTACAGCGTGAGCTATGAGAAAGCGCCACGCTTCCCGAAGGGAGAAAGGCGGACAGGTATCCGGTAAGCGGCAGG
GTCGGAACAGGAGAGCGCACGAGGGAGCTTCCAGGGGGAAACGCCTGGTATCTTTATAGTCCTGTCGGGTTTCGC
CACCTCTGACTTGAGCGTCGATTTTTGTGATGCTCGTCAGGGGGGCGGAGCCTATGGAAAAACGCCAGCAACGCG
GCCTTTTTACGGTTCCTGGCCTTTTGCTGGCCTTTTGCTCACATGTTCTTTCCTGCGTTATCCCCTGATTCTGTG
GATAACCGTATTACCGCCTTTGAGTGAGCTGATACCGCTCGCCGCAGCCGAACGACCGAGCGCAGCGAGTCAGTG
AGCGAGGAAGCGGAAGAGCGCCTGATGCGGTATTTTCTCCTTACGCATCTGTGCGGTATTTCACACCGCATATAT
GGTGCACTCTCAGTACAATCTGCTCTGATGCCGCATAGTTAAGCCAGTATACACTCCGCTATCGCTACGTGACTG
GGTCATGGCTGCGCCCCGACACCCGCCAACACCCGCTGACGCGCCCTGACGGGCTTGTCTGCTCCCGGCATCCGC
TTACAGACAAGCTGTGACCGTCTCCGGGAGCTGCATGTGTCAGAGGTTTTCACCGTCATCACCGAAACGCGCGAG
GCAGCTGCGGTAAAGCTCATCAGCGTGGTCGTGAAGCGATTCACAGATGTCTGCCTGTTCATCCGCGTCCAGCTC
GTTGAGTTTCTCCAGAAGCGTTAATGTCTGGCTTCTGATAAAGCGGGCCATGTTAAGGGCGGTTTTTTCCTGTTT
GGTCACTGATGCCTCCGTGTAAGGGGGATTTCTGTTCATGGGGGTAATGATACCGATGAAACGAGAGAGGATGCT
CACGATACGGGTTACTGATGATGAACATGCCCGGTTACTGGAACGTTGTGAGGGTAAACAACTGGCGGTATGGAT
GCGGCGGGACCAGAGAAAAATCACTCAGGGTCAATGCCAGCGCTTCGTTAATACAGATGTAGGTGTTCCACAGGG
TAGCCAGCAGCATCCTGCGATGCAGATCCGGAACATAATGGTGCAGGGCGCTGACTTCCGCGTTTCCAGACTTTA
CGAAACACGGAAACCGAAGACCATTCATGTTGTTGCTCAGGTCGCAGACGTTTTGCAGCAGCAGTCGCTTCACGT
TCGCTCGCGTATCGGTGATTCATTCTGCTAACCAGTAAGGCAACCCCGCCAGCCTAGCCGGGTCCTCAACGACAG
GAGCACGATCATGCGCACCCGTGGGGCCGCCATGCCGGCGATAATGGCCTGCTTCTCGCCGAAACGTTTGGTGGC
GGGACCAGTGACGAAGGCTTGAGCGAGGGCGTGCAAGATTCCGAATACCGCAAGCGACAGGCCGATCATCGTCGC
GCTCCAGCGAAAGCGGTCCTCGCCGAAAATGACCCAGAGCGCTGCCGGCACCTGTCCTACGAGTTGCATGATAAA
GAAGACAGTCATAAGTGCGGCGACGATAGTCATGCCCCGCGCCCACCGGAAGGAGCTGACTGGGTTGAAGGCTCT
CAAGGGCATCGGTCGAGATCCCGGTGCCTAATGAGTGAGCTAACTTACATTAATTGCGTTGCGCTCACTGCCCGC
TTTCCAGTCGGGAAACCTGTCGTGCCAGCTGCATTAATGAATCGGCCAACGCGCGGGGAGAGGCGGTTTGCGTAT
TGGGCGCCAGGGTGGTTTTTCTTTTCACCAGTGAGACGGGCAACAGCTGATTGCCCTTCACCGCCTGGCCCTGAG
AGAGTTGCAGCAAGCGGTCCACGCTGGTTTGCCCCAGCAGGCGAAAATCCTGTTTGATGGTGGTTAACGGCGGGA
TATAACATGAGCTGTCTTCGGTATCGTCGTATCCCACTACCGAGATATCCGCACCAACGCGCAGCCCGGACTCGG
TAATGGCGCGCATTGCGCCCAGCGCCATCTGATCGTTGGCAACCAGCATCGCAGTGGGAACGATGCCCTCATTCA
GCATTTGCATGGTTTGTTGAAAACCGGACATGGCACTCCAGTCGCCTTCCCGTTCCGCTATCGGCTGAATTTGAT
TGCGAGTGAGATATTTATGCCAGCCAGCCAGACGCAGACGCGCCGAGACAGAACTTAATGGGCCCGCTAACAGCG
CGATTTGCTGGTGACCCAATGCGACCAGATGCTCCACGCCCAGTCGCGTACCGTCTTCATGGGAGAAAATAATAC
TGTTGATGGGTGTCTGGTCAGAGACATCAAGAAATAACGCCGGAACATTAGTGCAGGCAGCTTCCACAGCAATGG
CATCCTGGTCATCCAGCGGATAGTTAATGATCAGCCCACTGACGCGTTGCGCGAGAAGATTGTGCACCGCCGCTT
TACAGGCTTCGACGCCGCTTCGTTCTACCATCGACACCACCACGCTGGCACCCAGTTGATCGGCGCGAGATTTAA
TCGCCGCGACAATTTGCGACGGCGCGTGCAGGGCCAGACTGGAGGTGGCAACGCCAATCAGCAACGACTGTTTGC
CCGCCAGTTGTTGTGCCACGCGGTTGGGAATGTAATTCAGCTCCGCCATCGCCGCTTCCACTTTTTCCCGCGTTT
TCGCAGAAACGTGGCTGGCCTGGTTCACCACGCGGGAAACGGTCTGATAAGAGACACCGGCATACTCTGCGACAT
CGTATAACGTTACTGGTTTCACATTCACCACCCTGAATTGACTCTCTTCCGGGCGCTATCATGCCATACCGCGAA
AGGTTTTGCGCCATTCGATGGTGTCCGGGATCTCGACGCTCTCCCTTATGCGACTCCTGCATTAGGAAGCAGCCC
AGTAGTAGGTTGAGGCCGTTGAGCACCGCCGCCGCAAGGAATGGTGCATGCAAGGAGATGGCGCCCAACAGTCCC

CCGGCCACGGGGCCTGCCACCATACCCACGCCGAAACAAGCGCTCATGAGCCCGAAGTGGCGAGCCCGATCTTCC
CCATCGGTGATGTCGGCGATATAGGCGCCAGCAACCGCACCTGTGGCGCCGGTGATGCCGGCCACGATGCGTCCG
GCGTAGAGGATCGAGATCTCGATCCCGCGAAATTAATACGACTCACTATAGGGGAATTGTGAGCGGATAACAATT
CCCCTCTAGAAATAATTTTGTTTAACTTTAAGAAGGAGATATACCATGGGCAGCAGCCATCATCATCATCATCAC
AGCAGCGGCCTGGTGCCGCGCGGCAGCCATATGGCTAGCATGACTGGTGGACAGCAAATGGGTCGCGGATCCGAA
TTCGAGCTCCGTCGACAAGCTTGCGGCCGCACTCGAGCACCACCACCACCACCACTGAGATCCGGCTGCTAACAA
AGCCCGAAAGGAAGCTGAGTTGGCTGCTGCCACCGCTGAGCAATAACTAGCATAACCCCTTGGGGCCTCTAAACG
GGTCTTGAGGGGTTTTTTGCTGAAAGGAGGAACTATATCCGGAT

3.6.2. *Ea*GK, Seq. 1

>AB005149.1 Exiguobacterium acetylicum gsk, orf2 genes, complete cds

GGTACCAGTCGCCATCTTGATCGGCTCGATCCTGCTTGCGAATAACATTCGGGACTTGGATAATGATAAGGTCAA
CGGACGAAAAACGATTGCCTGTCTCGTCGGACATCGCCGTGCTGTTTACGTCTTGATCGGATTTTTTGCGGCAGC
TGTCCTGTCTCTCATTATTGCTGTACTCGCATTCGAAGTCTCATGGTTTGCGTTACTCGCGTTGTTAAGTATTCC
ACTCATGATCAAAGCAGTCCGTCTCTTCTGGGAAGACTTGCCACCGGAAAAACTGATGCCTGGTATGGCACAGAC
TGGTAAAGTGAATACGATTTTCGGACTTTTACTCGCAATCAGTCTCGTGATCGCGAATATCTAATCTTAATTTAA
AGTAGCAGGCGATTCGCTTGCTGCTTTTTTTACGTGAAGATAGGGAACAGTTAGAAAAAAAGTACTTCGGTTGTC
ACTGATTTAAAATTTTACTATAATTGAAGCAATGTGCCTGAAAGATCAAACAAGGTCGGAAATCAGGAAATATGG
TTAGAGGTGGAACTGAAATGAATAAAATCGCGGTAATCGGAAAAGTATTCGTCGACATAAAAGGAACTTCGTTCG
CTCCTTTGCATAAGGATGCGAAAAACGTAGGAGACATCACGTTTTCAAATGGAGGAACAGGACGCAACGTAGCAC
AAAATCTAGCCGTCCTCGGGAATGAAGTTCGCTTTATCTCGACGGTTACGAATGATCAGATTGGCGTGGGAGTGC
TCGATGAGCTGAAATCCTACGGTGCGAATGTGGATCACGTCGAAATGTTAGAAGATCATGGAATGGGTATGTGGC
TAGCTGTCATGGATAACGAGGGTGACTTGCAAACATCGATCTCGAAACAACCGGATGCCAAGTTGCTCGAAGAGG
CGATTTTACGTCAATCGATCTATGCACTCGATGGAGTCGATGCCGTTGCAATCGATTTGGATTTGTCCGTCACGG
TCTTAGAACGTTTGATTCATTTATGTCGTAAGATGGAGTTGCCATTGTTTGGTGTTTGTGGTCACTTGAGCGTCA
TCGAACGAAATCGTCATCTGCTACAAGGGTTCACTGGATTCATTTGTAGCCGAGAAGAGGCTGAAATTCTGTCTG
ATCTATCGATCGTGACGGTCGAAGATGCGATTCATGTAGCAAATGAGCTAGCGAAAAAGGGCGCTCCGTTTACGG
TCGTGACGATGAGTGAACTGGGGGCGGTCTACGTTGATCGTCGTACGGCGACATCAGGTCACGTCGGAACGAAAA
AAGTGAAGGTTGTCGACTCAACGGGAGCAGGCGATTCCTTCTTCTCCGCAGTCTTGTCCGAATTGACACAGGAAA
AGTCAGCAGAAGAGGCTTTGAAGCTTGGTATGAAGGTCGCAGCAGAAGTCATCGCTTCAACAGAGAATGGACTCG
TTCCTGAAATGCTAGATGCTCTTCAATAAGTAGAAATGATAAAAGGGAGGTGGCGTCATGCCGATTCAACGAAGT
GCAATCATTTTACGAAACGAACAGGATGAAATTGCGTTGATCCGACGGGATAAACCGAACGAAACGTATTATGTC
TTTCCAGGCGGTGGGAAAGATGACGGAGAATCGTTAGAAGAAACAGCGATTCGCGAAGCACATGAAGAACTTGGC
ATCGACGTGGAGTTGACCGGTATTGCCGCCATCGTCCGTTTTAATGGATTCGATAATCCTTATTTTTGGGCGAAA
ACAATTGGTGGTCAGTTTGGCACGGGTACGGGTGAAGAATTCGAAGAGGAAGGATCGGGTTATACACCGGTCTGG
ATCAAGCGTTCGGAGCTACCTTCGTTGCCAATTCGTCCACCGTCACTTGCCAAACAACTGGCAGAAATGACAGAA
CCATTCTATGAATTAATCCTTTCTGAAAACGAATGAGGTTTTAGTTGAAATAAAATGGGAAAAGTATATTAAAAC
AATGAGGTGGTCTTCGACTGCCTCTTTTTCATGAAGGAGAGATGACGATGGCGCATTCACGCGATCCAAAAGGGC
TTGCCCTTAACTTAAAGCAGCGAATGTCCGATCATAATATCACGGATTATGCAGGGACACTCGCCTATTACTGGT
TTTTATCCATTTTCCCAGGAATCATTTTCGTCATTTCGGTCTTATCATTCTTTGATATCGATCGTCAGACACTCG
AGTCACAAATTCGGGACCTAGCGCCAGGCGGAGCCGTTAATACGTTCACGGATACAATTTTCCAAGCCATCAAAG
AACCGCAGGGTGGTCTGTTATCGATCGGTGCGATTCTTGCTGTCTGGTCCGCTTCCAAAGGAGTCGATCGTTTGA
TCACGACAGCAAACCATGCCTATGGTGACTTCTCTCCCCGTGGTTTTGTCGCAGCGCGTGGGATTGCCTTGTTAC
TCACGATCGTGCTCGGAGTCGGGATGTTGTTGCTGATCGTCTTGAATGTGCTCGGTGGTCCGATCATTACGTATC
TTGCCAACTTCGTGTTGCCGATCGATATGGGGCAAAAAATCCTACTGACGGTCTTACGATATGTCGTATCGACGA
TTCTATTGATTGGTATCCTGTCGATTTTTTACCGCGTCGCACCAAAGCGCCCGATTACATTTAAAGAGGCAATTC
CAGGAGCCGTCTTTGGTGTCATCGTCTGGCAATTATTATCGGTTGGATTCGGATTTTACGTCTCGAACTTCTCGA
ACTACAATCAGACCTACGGTTCGCTCGGTAGTGTCGTCATCTTGTTATTATGGCTCTACTTCACAGGGCTGATCA
TCTTGCTCGGTTCTGAATTGAACGCATCATGGGAACGGTTCATGAAAAAAGCGGATCC

>sp|O24767|INGK_EXIAC Guanosine-inosine kinase OS=Exiguobacterium acetylicum OX=41170 GN=gsk PE=1 SV=1 + N terminal His$_6$-tag

HHHHHHMNKIAVIGKVFVDIKGTSFAPLHKDAKNVGDITFSNGGTGRNVAQNLAVLGNEVRFISTVTNDQIGVGV
LDELKSYGANVDHVEMLEDHGMGMWLAVMDNEGDLQTSISKQPDAKLLEEAILRQSIYALDGVDAVAIDLDLSVT
VLERLIHLCRKMELPLFGVCGHLSVIERNRHLLQGFTGFICSREEAEILSDLSIVTVEDAIHVANELAKKGAPFT
VVTMSELGAVYVDRRTATSGHVGTKKVKVVDSTGAGDSFFSAVLSELTQEKSAEEALKLGMKVAAEVIASTENGL
VPEMLDALQ

3.6.3. *Sc*AK, Seq. 2

>Z49605.1 S.cerevisiae chromosome X reading frame ORF YJR105w

TATAAATTAATTATGTTTTATTTGTTTGCGCGATTGCTTTCTTGCCTGTTTTTCCGCAACATAGTTACAGCTAAA
CATTTGCCCAAAACCATCTTCTTATATATATATCTGCGATGGCGAGCCCAGCGGAAGGGATGTCCGCTTACTAAT
TCCGACACACCGGTTTAACCCCCCGGCGTGCTGGGGTGGGTGCCCCTAAGGGAGCGGGTTCTGTACTTCCAGTAA
GCGGCATTTGCGCTGTCATCGCCTTATCGAACCCGCTACTGAGATCATGTCCTGAGTGGGTGAGTCGCACGCCCA
ATCGGCTGCACTTCAGAGATACTTACGTCCAATTTCGCATCATGCCAATATTACCTGTGGAAGTTGAAACTTTTT
TTGCAGATGCCGATAATTTTCACATGGATGAGTCGCAGTGTCGTCGTACGTGATGAGGGCAGGCTTGAAAATTAT
TCTTCGAGCCCAATTCTCTTTACTTATAGGCATGCCATGTCTAATTAGGTAGTCATCTCAGTTTATCTTGTGCAG
GGTAGAACCAAAGATAACAGCAAAAGAAAGAGCAAGCAATTATGACCGCACCATTGGTAGTATTGGGTAACCCAC
TTTTAGATTTCCAAGCCGACGTCACGGCTGAATACCTGGCCAAGTATTCTCTAAAGGAAAACGACGCAATTTTGG
TCGATGCCAAATCAGGCGATGCTAAGATGGCTATTTTTGACGAGCTCTTACAGATGCCAGAAACAAAGCTTGTTG
CAGGTGGTGCTGCTCAAAACACTGCTAGAGGGGCAGCATACGTTTTGGGCGCCGGCCAGGTGGTGTACTTCGGTT
CCGTCGGTAAGGACAAGTTCAGCGAGAGATTGCTTAACGAAAACGAAAAAGCTGGTGTCAAGTCTATGTACCAAG
TTCAAAATGATATTGGTACCGGTAAGTGTGCCGCATTAATCACTGGCCATAACCGGTCCTTGGTCACTGACTTGG
GTGCTGCCAATTTCTTTACTCCAGACCACTTGGACAAGCATTGGGACTTGGTCGAAGCAGCTAAGCTCTTCTACA
TCGGTGGTTTCCACTTGACCGTGTCTCCAGACGCTATCGTTAAGTTGGGCCAACATGCTAAAGAGAACAGCAAAC
CTTTCGTGTTGAACTTTAGTGCTCCTTTCATTCCTCATGTCTTCAAAGACGCATTGGCCAGAGTTTTGCCTTATG
CTACCGTCATCATCGCTAACGAATCGGAGGCCGAAGCCTTTTGCGACGCCTTCCAATTAGACTGTGCCAACACTG
ATTTGGAAGCTATTGCTCAAAGAATTGTCAAGGACTCTCCAGTTGAAAAGACTGTCATCTTCACCCACGGTGTCG
AACCAACAGTGGTCGTGTCCTCCAAGGGTACCAGCACATATCCAGTCAAACCTTTGGACTCTTCTAAGATCGTCG
ACACCAACGGTGCTGGTGACGCCTTCGCTGGTGGCTTTATGGCTGGGTTGACTAAAGGTGAAGATTTGGAAACCT
CTATTGACATGGGTCAATGGCTAGCTGCTTTGTCTATTCAAGAAGTTGGTCCCTCTTACCCTTCCGAAAAAATAT
CTTACTCTAAATAGATTCTTCTTACAATATAATAGAAATAAAATATACTCTACGTGTTCCCTCTAGCTTTTCATT
CGTAGACATGTTTACCGCTTGTTCTTAAACCAAATAAGCCACCGTTACCACAATAAGGAAAAGAGGCGCTATAAA
GTACAGCTTCAAGAAAAAAGGACAAGCAGCGCTAAGAACAAAATACTCGTTCATCCTCTCCACATACGGCCGTAC
ACAAGCAACAATAACACAATAAT

>sp|P47143|ADK_YEAST Adenosine kinase OS=Saccharomyces cerevisiae (strain ATCC 204508 / S288c) OX=559292 GN=ADO1 PE=1 SV=1 + N terminal His_6-tag

HHHHHHMTAPLVVLGNPLLDFQADVTAEYLAKYSLKENDAILVDAKSGDAKMAIFDELLQMPETKLVAGGAAQNT
ARGAAYVLGAGQVVYFGSVGKDKFSERLLNENEKAGVKSMYQVQNDIGTGKCAALITGHNRSLVTDLGAANFFTP
DHLDKHWDLVEAAKLFYIGGFHLTVSPDAIVKLGQHAKENSKPFVLNFSAPFIPHVFKDALARVLPYATVIIANE
SEAEAFCDAFQLDCANTDLEAIAQRIVKDSPVEKTVIFTHGVEPTVVVSSKGTSTYPVKPLDSSKIVDTNGAGDA
FAGGFMAGLTKGEDLETSIDMGQWLAALSIQEVGPSYPSEKISYSK

3.6.4. *Sc*GMPK, Seq. 3

```
>L04683.1 Yeast guanylate kinase (GUK1) gene, complete cds

AAGCTTGGAAACACATAAAAAAGAAAAAAAAAAAATACATTTAGCATAAATGGATACACAATGGCGTTTCTTTTTA
GTTGATAACTACAGTTTACTTCGGATAAAGATTAGTAAACCTTTGCGTTATGTCCCGTCCTATCGTAATTTCTGG
CCCAAGTGGTACAGGTAAATCTACACTGTTGAAGAAATTGTTCGCTGAATACCCAGATTCTTTCGGGTTTAGTGT
TTCATCCACTACTAGAACCCCAAGAGCTGGCGAAGTAAACGGTAAGGACTATAACTTTGTCTCCGTAGATGAATT
CAAATCTATGATTAAGAACAATGAATTCATTGAATGGGCGCAATTCTCCGGTAACTACTATGGTAGTACTGTCGC
TTCCGTCAAACAAGTCAGTAAATCTGGTAAGACTTGTATTTTAGATATTGATATGCAGGGTGTCAAATCTGTCAA
GGCTATCCCAGAGTTAAATGCCAGGTTTTTGTTTATTGCTCCACCATCGGTCGAGGATTTGAAAAAAAGATTAGA
AGGTAGAGGTACGGAGACCGAAGAATCCATCAACAAGAGGTTAAGCGCCGCTCAAGCTGAATTGGCATATGCTGA
GACAGGTGCCCATGACAAAGTTATTGTCAATGATGATTTGGACAAGGCCTACAAGGAATTGAAGGATTTTATCTT
TGCAGAAAAATGATGTAGCCCTATATAGACATTACTAAGTATGTACCTGGTAGGAGAGTCCTGTCGCAAAGCGAC
AAAACGTCCAATTATTCAATTAATATAGTGTAAAAGTTCTCAACGGGCTTATGCTAGTTTTTTTTGTTAGTAAGC
GCTACGACGACTAGAACCATCTCTTGAATTTCCAAGTGCCAAAATCAATGACCACGGATACTGTGGCCAGGAATC
TGTTGGTTGGTCATCCTCAAGATCT

>sp|P15454|KGUA_YEAST Guanylate kinase OS=Saccharomyces cerevisiae (strain
ATCC 204508 / S288c) OX=559292 GN=GUK1 PE=1 SV=2 + N terminal His6-tag

HHHHHHMSRPIVISGPSGTGKSTLLKKLFAEYPDSFGFSVSSTTRTPRAGEVNGKDYNFVSVDEFKSMIKNNEFI
EWAQFSGNYYGSTVASVKQVSKSGKTCILDIDMQGVKSVKAIPELNARFLFIAPPSVEDLKKRLEGRGTETEESI
NKRLSAAQAELAYAETGAHDKVIVNDDLDKAYKELKDFIFAEK
```

5. Supporting References

(1) Tasnádi, G.; Jud, W.; Hall, M.; Baldenius, K.; Ditrich, K.; Faber, K. Evaluation of Natural and Synthetic Phosphate Donors for the Improved Enzymatic Synthesis of Phosphate Monoesters. *Adv. Synth. Catal.* **2018**, *360* (12), 2394–2401. https://doi.org/10.1002/adsc.201800306.

(2) Fehlau, M.; Kaspar, F.; Hellendahl, K. F.; Schollmeyer, J.; Neubauer, P.; Wagner, A. Modular Enzymatic Cascade Synthesis of Nucleotides Using a (d)ATP Regeneration System. *Front. Bioeng. Biotechnol.* **2020**, *8*, 854. https://doi.org/10.3389/fbioe.2020.00854.

(3) Benčić, P.; Keppler, M.; Kuge, M.; Qiu, D.; Schütte, L. M.; Häner, M.; Strack, K.; Jessen, H. J.; Andexer, J. N.; Loenarz, C. Non-canonical Nucleosides: Biomimetic Triphosphorylation, Incorporation into mRNA and Effects on Translation and Structure. *FEBS J* **2023**, *290* (20), 4899–4920. https://doi.org/10.1111/febs.16889.

(4) Meng, Q.; Benckendorff, C.; Morrill, C.; Zhuo, Y.; Egerström, A.; Ní Cheallaigh, A.; Derrington, S. R.; Obexer, R.; Ortmayer, M.; Levy, C. W.; Finnigan, J. D.; Charnock, S. J.; Turner, N. J.; Miller, G. J.; Lovelock, S. L. Enzymatic Synthesis of Key RNA Therapeutic Building Blocks Using Simple Phosphate Donors. *Nat. Commun.* **2025**. https://doi.org/10.1038/s41467-025-67366-4.

(5) Wu, J.; Feng, L.; Xiao, C. Method for Synthesizing 3 ’-Methoxyguanosine Diphosphate through Multi-Enzyme Cascade Reaction and Application of 3’-Methoxyguanosine Diphosphate. CN116716367A, September 8, 2023.

(6) Huang, Y.; Li, J.; Zhang, Y.; Zhang, B.; Bian, Q.; Sun, F. Synthesis Method of 3 “-Amino-2”, 3 ’-Dideoxyguanosine 5’-Triphosphate. CN117143942A, December 1, 2023.

(7) Hennig, M.; Scott, L. G.; Sperling, E.; Bermel, W.; Williamson, J. R. Synthesis of 5-Fluoropyrimidine Nucleotides as Sensitive NMR Probes of RNA Structure. *J. Am. Chem. Soc.* **2007**, *129* (48), 14911–14921. https://doi.org/10.1021/ja073825i.

(8) Lee, T. T.; Momparler, R. L. Enzymatic Synthesis of 5-Azacytidine 5′-Triphosphate from 5-Azacytidine. *Anal. Biochem.* **1976**, *71* (1), 60–67. https://doi.org/10.1016/0003-2697(76)90011-7.
(9) Chan, K. K. Method for Use in Synthesizing β-Thymidine. WO2018196235A1, November 1, 2018.
(10) Huang, Y.; Li, J.; Zhang, Y.; Wang, Q.; Ye, J.; Zhang, B.; Bian, Q.; Sun, F.; Gu, J. Method for Preparing Pseudouridine-5 ’-Triphosphate, Enzyme Combination and Application of Enzyme Combination. CN117265043A, December 22, 2023.
(11) Huang, Y.; Li, J.; Zhang, Y.; Zhang, B.; Bian, Q.; Sun, F. Method for Preparing N1-Methyl Pseudouridine Triphosphate. CN117587086A, February 23, 2024.
(12) Da Costa, C. P.; Fedor, M. J.; Scott, L. G. 8-Azaguanine Reporter of Purine Ionization States in Structured RNAs. *J. Am. Chem. Soc.* **2007**, *129* (11), 3426–3432. https://doi.org/10.1021/ja067699e.
(13) Jong, A.; Yeh, Y.; Ma, J. J. Characteristics, Substrate Analysis, and Intracellular Location of *Saccharomyces Cerevisiae* UMP Kinase. *Arch. Biochem. Biophys.* **1993**, *304* (1), 197–204. https://doi.org/10.1006/abbi.1993.1339.
(14) Pfeiffer, M.; Krammer, L.; Zöhrer, J.; Breinbauer, R.; Nidetzky, B. Integrated Chemoenzymatic Synthesis of the mRNA Vaccine Building Block N1-Methylpseudouridine Triphosphate. *Angew. Chem. Int. Ed.* **2025**, *64* (34), e202506330. https://doi.org/10.1002/anie.202506330.
(15) Pfeiffer, M.; Nidetzky, B. Reverse C-Glycosidase Reaction Provides C-Nucleotide Building Blocks of Xenobiotic Nucleic Acids. *Nat. Commun.* **2020**, *11* (1), 6270. https://doi.org/10.1038/s41467-020-20035-0.
